# Goblet Cell Derived RELM-β Recruits CD4^+^ T Cells during Infectious Colitis to Promote Protective Intestinal Epithelial Cell Proliferation

**DOI:** 10.1371/journal.ppat.1005108

**Published:** 2015-08-18

**Authors:** Kirk S. B. Bergstrom, Vijay Morampudi, Justin M. Chan, Ganive Bhinder, Jennifer Lau, Hyungjun Yang, Caixia Ma, Tina Huang, Natasha Ryz, Ho Pan Sham, Maryam Zarepour, Colby Zaph, David Artis, Meera Nair, Bruce A. Vallance

**Affiliations:** 1 Division of Gastroenterology, Department of Pediatrics, Child and Family Research Institute, Vancouver, Canada; 2 Biomedical Research Centre, University of British Columbia, Vancouver, Canada; 3 Jill Roberts Institute for Research in Inflammatory Bowel Disease, Joan and Sanford Weill Department of Medicine, West Cornell Medical College, Cornell University, New York, New York, United States of America; 4 Division of Biomedical Sciences, University of California, Riverside, Riverside, California, United States of America; University of California Davis, UNITED STATES

## Abstract

Enterohemorrhagic *Escherichia coli* and related food and waterborne pathogens pose significant threats to human health. These attaching/effacing microbes infect the apical surface of intestinal epithelial cells (IEC), causing severe diarrheal disease. Colonizing the intestinal luminal surface helps segregate these microbes from most host inflammatory responses. Based on studies using *Citrobacter rodentium*, a related mouse pathogen, we speculate that hosts rely on immune-mediated changes in IEC, including goblet cells to defend against these pathogens. These changes include a CD4^+^ T cell-dependent increase in IEC proliferation to replace infected IEC, as well as altered production of the goblet cell-derived mucin Muc2. Another goblet cell mediator, REsistin-Like Molecule (RELM)-β is strongly induced within goblet cells during *C*. *rodentium* infection, and was detected in the stool as well as serum. Despite its dramatic induction, RELM-β’s role in host defense is unclear. Thus, wildtype and RELM-β gene deficient mice (*Retnlb* ^-/-^) were orally infected with *C*. *rodentium*. While their *C*. *rodentium* burdens were only modestly elevated, infected *Retnlb* ^-/-^ mice suffered increased mortality and mucosal ulceration due to deep pathogen penetration of colonic crypts. Immunostaining for Ki67 and BrDU revealed *Retnlb* ^-/-^ mice were significantly impaired in infection-induced IEC hyper-proliferation. Interestingly, exposure to RELM-β did not directly increase IEC proliferation, rather RELM-β acted as a CD4^+^ T cell chemoattractant. Correspondingly, *Retnlb* ^-/-^ mice showed impaired CD4^+^ T cell recruitment to their infected colons, along with reduced production of interleukin (IL)-22, a multifunctional cytokine that directly increased IEC proliferation. Enema delivery of RELM-β to *Retnlb* ^-/-^ mice restored CD4^+^ T cell recruitment, concurrently increasing IL-22 levels and IEC proliferation, while reducing mucosal pathology. These findings demonstrate that RELM-β and goblet cells play an unexpected, yet critical role in recruiting CD4^+^ T cells to the colon to protect against an enteric pathogen, in part via the induction of increased IEC proliferation.

## Author Summary

Food and water-borne bacterial pathogens such as enterohemorrhagic *Escherichia coli* (EHEC) target the epithelial cells that line the inner surface of their host’s intestines, causing inflammation and diarrhea. While professional immune cells including T lymphocytes are well known for promoting host defense, we hypothesized that as the cells in closest contact with these bacterial pathogens, intestinal epithelial cells also play an active and essential role in protecting the host during infection. Infecting mice with *Citrobacter rodentium*, a mouse specific relative of EHEC, we noted a dramatic upregulation in the expression and secretion of the mediator RELM-β by a subset of epithelial cells called goblet cells. Compared to wildtype mice, mice lacking RELM-β showed less epithelial cell proliferation and suffered significantly more intestinal damage during infection. Rather than directly causing epithelial cell proliferation, we found RELM-β instead recruited T lymphocytes to the infected intestine. Upon reaching the intestine, the T lymphocytes produced the cytokine interleukin-22, which directly increased epithelial cell proliferation. Taken together, these findings indicate that epithelial/goblet cells play a critical role in orchestrating the host response to an intestinal pathogen, by recruiting T lymphocytes and by promoting epithelial proliferation to limit the intestinal damage suffered during infection.

## Introduction

The enteric bacterial pathogens enterohemorrhagic *Escherichia coli* (EHEC) and enteropathogenic *E*. *coli* (EPEC) are important causes of infectious diarrhea. These food and waterborne pathogens infect intestinal epithelial cells (IEC) using a Type III secretion system (T3SS) [1]. Their infection leads to characteristic attaching and effacing (A/E) lesions on IEC, as well as diarrhea and transient enteritis or colitis in humans [1]. Exactly how the host defends against these A/E pathogens is poorly understood, largely because their luminal location segregates and protects them from most inflammatory and immune effector mechanisms. Instead, we and others have hypothesized that host defense against these microbes relies largely on immune mediated changes in the intestinal epithelium. In fact, several *in vitro* studies have shown that IEC actively promote “host resistance” to A/E pathogens by producing factors that recruit inflammatory/immune cells to the infected intestine, and by upregulating their expression of antimicrobial peptides to directly kill A/E bacteria [2–5]. However the efficacy of IEC-driven responses in clearing these pathogens is unclear, raising the question of whether infected hosts also promote IEC responses that help the host “tolerate” these infections, by limiting intestinal tissue damage to ensure the host’s survival.

Unfortunately the human specificity of EPEC and EHEC has limited our ability to study host responses against these microbes. *Citrobacter rodentium*, a natural A/E pathogen of mice has been widely used to model EPEC and EHEC infections, as well as study the host immune responses that develop against these pathogens [6, 7]. We and others have shown that CD4^+^ T cells are recruited to the infected intestine, where they drive a mixed Th1/Th17/Th22 immune response that promotes host defense, by limiting *C*. *rodentium* burdens [8–11]. Moreover *C*. *rodentium* infection leads to significant increases in IEC-based expression of antimicrobial proteins and chemokines, as well as dramatic elongation (hyperplasia) of colonic crypts due to increased IEC proliferation. We recently showed these hyperplastic crypts appear less susceptible to infection by *C*. *rodentium* [12] whereas the highly susceptible *Rag1* deficient (^-/-^) mice (lacking T and B cells) are severely impaired in developing infection-induced IEC hyper-proliferation. Notably, reconstitution of *Rag1* ^-/-^ mice with CD4^+^, but not CD8^+^ T cells largely restored the IEC hyper-proliferative response during infection [13]. While IEC hyper-proliferation has been primarily seen as a characteristic pathology of *C*. *rodentium* infection, it may also reflect one mechanism by which CD4^+^ T cells promote host defense against A/E pathogens, although exactly how this response benefits the infected host remains controversial.

Increased proliferation and shedding of infected IEC are unlikely to clear the intestine of invading microbes, and also have detrimental effects on the host, such as limiting the maturation and differentiation of IEC including goblet cells, thereby limiting mucin production as well as ion transport in the colon [12–16]. Even so, increasing IEC turnover likely limits the potential for lumen dwelling microbes to escape the intestine and go systemic, as well as ensuring the replacement of IEC damaged by the pathogen, or by the host’s own inflammatory response. Thus the immune-mediated increase in IEC proliferation may fall under the new designation of “tolerance responses” that limit the pathology suffered by the host during infection [17]. Other potential tolerance responses described during *C*. *rodentium* infection include TLR2-dependent signaling, which rather than impacting *C*. *rodentium* burdens, was shown to limit mucosal damage as well as protect IEC barrier function during infection [18, 19]. In fact, tolerance responses may be selected for when dealing with intestinal pathogens since resistance responses aimed at killing pathogens may inadvertently deplete commensal microbes. We recently demonstrated this effect in mice lacking the negative regulator of TLR/IL-1R signaling termed SIGIRR [20]. *Sigirr-* ^*/-*^ mice proved highly susceptible to *C*. *rodentium* infection despite developing an exaggerated antimicrobial response, because rather than killing the pathogen, their host response caused a rapid depletion of commensal microbes, thus reducing colonization resistance against *C*. *rodentium* [20].

Aside from undergoing increased proliferation, secretory IEC such as goblet cells can also release mediators that promote host defense. For example, goblet cells produce and release the polymeric gel-forming mucin Muc2 into the intestinal lumen, where it hydrates and forms the protective mucus layer that overlies the IEC [21, 22]. Suspecting that Muc2 would play a protective role in this model, we previously infected wildtype mice as well as mice lacking intestinal mucus (*Muc2* ^*-/-*^). The mucus barrier not only delayed *C*. *rodentium* infection, but mucin production increased during infection, acting to “flush” loosely adherent pathogens away from the mucosal surface [23]. Intestinal goblet cells also produce other mediators, including Resistin-like molecule (RELM)-β [24, 25]. RELM-β belongs to a family of cysteine-rich secretory molecules initially described to control insulin resistance in rodents [26, 27]. Interestingly, RELM proteins have been shown to modulate inflammation and wound healing processes [24, 28, 29]. RELM-β is produced solely by goblet cells, and is induced in the intestines of germfree mice following their colonization by commensal bacteria [30]. RELM-β expression is also strongly induced in mouse models of spontaneous ileitis [31] and during dextran-sodium sulfate driven colitis [24, 32], where it appears to worsen intestinal inflammation by stimulating macrophage production of pro-inflammatory cytokines such as TNFα, IL-6, and RANTES. Moreover, RELM-β has been shown to modulate host defense during helminth parasite infections (*Trichuris muris*, *Nippostrongylus brasiliensis*), by impacting on parasite viability and on CD4^+^ T cell cytokine responses during infection [33, 34].

Despite these findings, the actions of RELM-β within the GI tract remain controversial, and are largely unexplored during enteric bacterial infections. To better define its function, we tested whether RELM-β contributes to the host response to *C*. *rodentium*. Infection dramatically increased RELM-β levels within colonic goblet cells as well as in the stool and sera of mice. Although mice lacking the RELM-β gene (*Retnlb* ^-/-^) carried roughly similar intestinal and systemic pathogen burdens to wildtype mice, they proved highly susceptible to *C*. *rodentium*, suffering exaggerated mucosal injury, in concert with impaired proliferation and replacement of infected IEC. Considering that CD4+ T cells are required for the increased IEC proliferative response during infection [13], we tested if *Rag1* ^-/-^ mice were impaired in RELM-β production, but instead found it was strongly expressed in their colons during infection. Instead we determined that RELM-β functions as a chemoattractant for CD4^+^ T cells, and that infected *Retnlb* ^-/-^ mice suffered a significant delay in CD4^+^ T cell recruitment to the intestine, along with reduced levels of interleukin (IL)-22, a cytokine that can directly increase IEC proliferation. Moreover, enema delivery of RELM-β to *Retnlb* ^-/-^ mice restored CD4^+^ T cell recruitment, elevating IL-22 levels and IEC proliferation, while reducing mucosal pathology. These results demonstrate that RELM-β and goblet cells play a novel host protective role during infectious colitis, accelerating the recruitment of CD4^+^ T cells and the promotion of IEC proliferation within the infected intestine, thereby limiting infection-associated tissue pathology.

## Results

### RELM-β expression is strongly induced during *C*. *rodentium* infection

**Fig 1:**
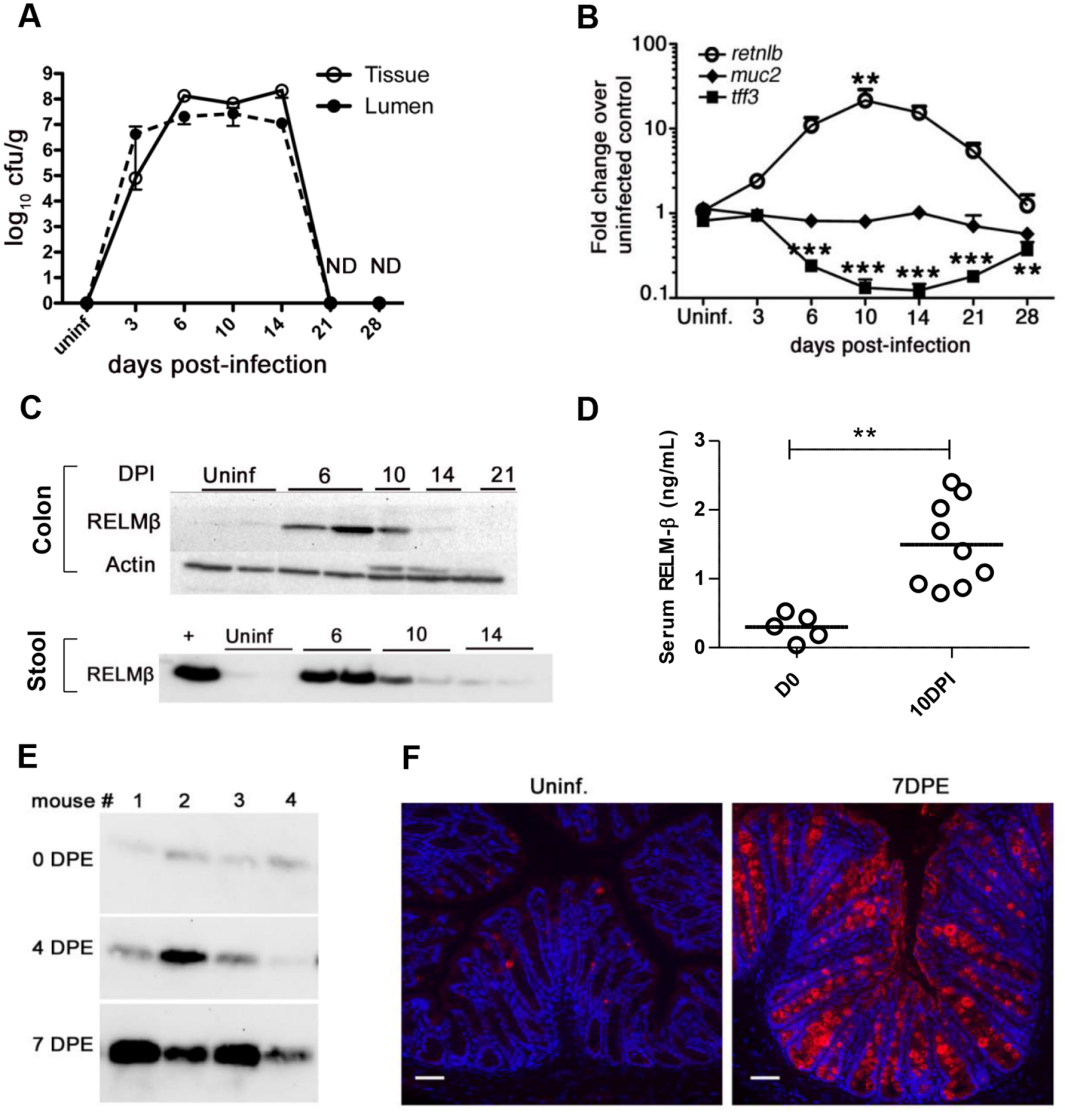
Dynamics of RELM-β expression during *C*. *rodentium* infection. (A) Enumeration of tissue adherent or luminal *C*. *rodentium* following oral gavage. Results show mean values of 3–4 mice/group. (B) qPCR analysis of RELM-β, Muc2, and TFF3 mRNA levels in the distal colons of C57BL/6 mice. Results show mean values of 3–4 mice/group, **P = 0.0014 vs. uninfected; ***P < 0.0001 vs. uninfected. (C) Western blots of RELM-β protein in colonic tissues and stool lysates of uninfected or infected mice. Lanes show data from single mice, representative of >4 mice/group. + indicates rRELM-β positive control. (D) Presence of RELM-β in the serum as detected by ELISA in uninfected or infected (10 DPI) mice. Each data point represents 1 mouse. Error bars = SEM. ** *P* < 0.001. (E) Western blot of RELM-β within stool of mice 7 days following their natural exposure to *C*. *rodentium* infected mice. Each lane follows 1 animal over time. (F) Representative immunofluorescent staining of RELM-β in tissues of uninfected or 7 days post exposure (DPE) mice following natural transmission. n = 4/group. Scale bars = 100 μm. Original magnification = 200X. All results are representative of at least 2 independent experiments.

Recent studies have shown that infection of the murine intestine by *C*. *rodentium* induces the resident goblet cells to strongly express RELM-β [30, 35], however the duration of this response and whether RELM-β was secreted by the goblet cells was not examined. To test this, C57BL/6 mice were infected with *C*. *rodentium* and colonic tissues, stool and serum were analyzed. Between 6 and 10 DPI when bacterial burdens were sustained at CFU levels up to 10^8^/gram tissue (Fig 1A), we noted a dramatic increase in *Retnlb* gene transcript levels in the distal colon that remained elevated until the infection was cleared (21–28 DPI) (Fig 1A and 1B). When other goblet cell-specific mediators were assessed over this time course, Muc2 gene transcript levels remained fairly stable whereas trefoil factor (TFF) 3 transcripts decreased at 10 and 14 DPI as described previously [12], returning to near baseline levels by 28 DPI (Fig 1B). We also assessed RELM-β protein levels, revealing a corresponding increase in RELM-β expression in colon tissues at 6 DPI (Fig 1C). In addition, significant levels of RELM-β protein were also detected in stool samples isolated at 6 and 10 DPI (Fig 1C) as well as in the sera (Fig 1D) indicating that RELM-β is released from the goblet cells during infection. At later time points, RELM-β protein levels decreased from their peak (Fig 1C), likely reflecting increasing inflammatory damage in the colon, as well as accelerated IEC/goblet cell turnover rates and immaturity at these later stages of *C*. *rodentium* infection [13]. These data thus reveal a highly dynamic goblet cell response to *C*. *rodentium*, with RELM-β showing a distinct induction during infection.

To ensure the dramatic induction of RELM-β seen during infection was not simply due to the large oral dose of *C*. *rodentium*, we also tested a natural infection model by co-housing previously unexposed C57BL/6 mice with *C*. *rodentium-*infected mice. At day 7 post-exposure (DPE) when *Citrobacter* infection is well established in the previously naive mice (S1A Fig), we noted a significant ((40-fold) P < 0.0001) induction in *Retnlb* gene expression (S1B Fig). Western blotting of stool samples revealed a major increase in secreted RELM-β protein levels at 7 DPE (Fig 1E), while immunostaining confirmed both RELM-β induction and its specificity to goblet cells (Fig 1F).

### RELM-β deficiency leads to increased mortality and mucosal damage during infection

**Fig 2:**
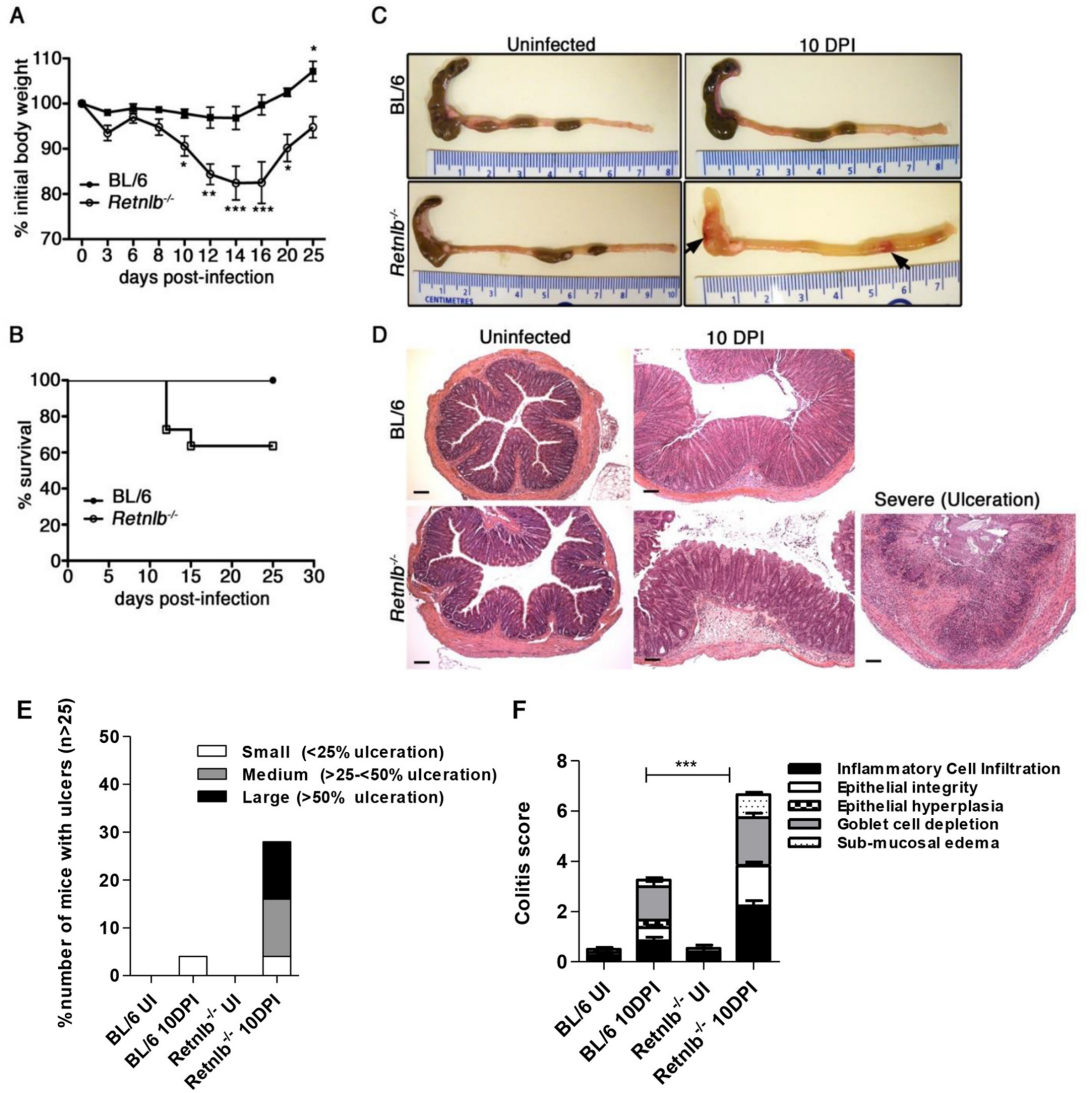
*Retnlb* ^*-/-*^ mice are highly susceptible to *C*. *rodentium*-induced colitis. (A) Body weights following infection of C57BL/6 and *Retnlb* ^-/-^ mice. Error bars = SEM. *P < 0.05; * *P* < 0.05 *** *P* < 0.0001 (vs. 0 DPI). Results are representative of at least 3 experiments, n = 3–6/group. (B) Infection survival curve of C57BL/6 mice and *Retnlb* ^-/-^ mice. Starting numbers (i.e at 0 DPI) were WT, n = 9; and *Retnlb* ^-/-^, n = 11. Results are representative of 3 experiments. (C) Resected colons and ceca of uninfected and 10 DPI C57BL/6 and *Retnlb* ^*-/-*^ mice. Results are representative of ≥ 4 infections, n = 3–6/group. (D) Representative H&E-stained colon sections from C57BL/6 and *Retnlb* ^*-/-*^ mice at 10 DPI (n ≥10/group). (E) Ulcer frequency and size in C57BL/6 vs. *Retnlb* ^*-/-*^ mice at 10 DPI. Bars show average mean values of ≥ 3 independent experiments, each with n = 2–4 mice/group. (F) Histopathology scoring of C57BL/6 vs. *Retnlb* ^-/-^ mice (n ≥ 10 per group). *** *P* < 0.0001. All error bars = SEM.

We next examined mice genetically deficient in RELM-β (*Retnlb-* ^/-^ mice), and consistent with previous reports [24, 32] found no evidence of overt or spontaneous disease development under uninfected conditions. Similarly, upon examining the intestinal tissues of uninfected C57BL/6 and *Retnlb-* ^/-^ mice, we noted no overt pathology or other baseline differences between strains. In contrast, when we infected *Retnlb* ^-/-^ mice, we found they suffered a significant drop (15–20%) from their initial body weights that lasted until at least 16 DPI, whereas infected wildtype mice maintained their weight throughout the infection (Fig 2A). Furthermore, during each round of infections, on average 30–40% of *Retnlb* ^-/-^mice exhibited such significant morbidity (became moribund) that they required euthanization (Fig 2B). While infected C57BL/6 mice suffered widespread but modest intestinal macroscopic pathology (as typical for this strain) at 8 and 10 DPI, infected *Retnlb* ^-/-^ mice displayed grossly swollen colons completely devoid of stool contents, as well as overt mucosal bleeding and patchy ulceration in both the cecum and colon (Fig 2C, arrows). Histologic analysis and pathology scoring of the colons of *Retnlb-* ^/-^ mice revealed marked epithelial disruption as well as significant but patchy influx of inflammatory cells in the infected mucosa, with some regions also showing significant submucosal edema and altered mucosal architecture (Fig 2D and 2F). The most severe phenotype seen in the *Retnlb* ^-/-^ mice involved pan-ulceration and massive dropout of crypts, leaving the entire tissue cross-section severely necrotic (Fig 2D and 2E). The intestinal pathology seen in *Retnlb* ^-/-^ mice at 8 DPI (S2A and S2B Fig) was similar to that seen at 10 DPI (Fig 2D), and exaggerated pathology was also observed in the ceca of infected *Retnlb* ^-/-^ mice (S2C and S2D Fig). In contrast, infected C57BL/6 mice suffered significantly (*P* < 0.0001) less colonic pathology, characterized by modest but widespread inflammatory cell infiltration and crypt hyperplasia (Fig 2D and 2F). Notably, the exaggerated pathology scores suffered by infected *Retnlb* ^-/-^ mice compared to C57BL/6 mice were also observed following their co-housing suggesting their susceptibility was due to their genotype rather than an aberrant microbiome. These results thus show that loss of RELM-β renders mice significantly more susceptible to *C*. *rodentium*-induced colitis.

### RELM-β deficiency is associated with deeper penetration of crypts by *C*. *rodentium*

**Fig 3:**
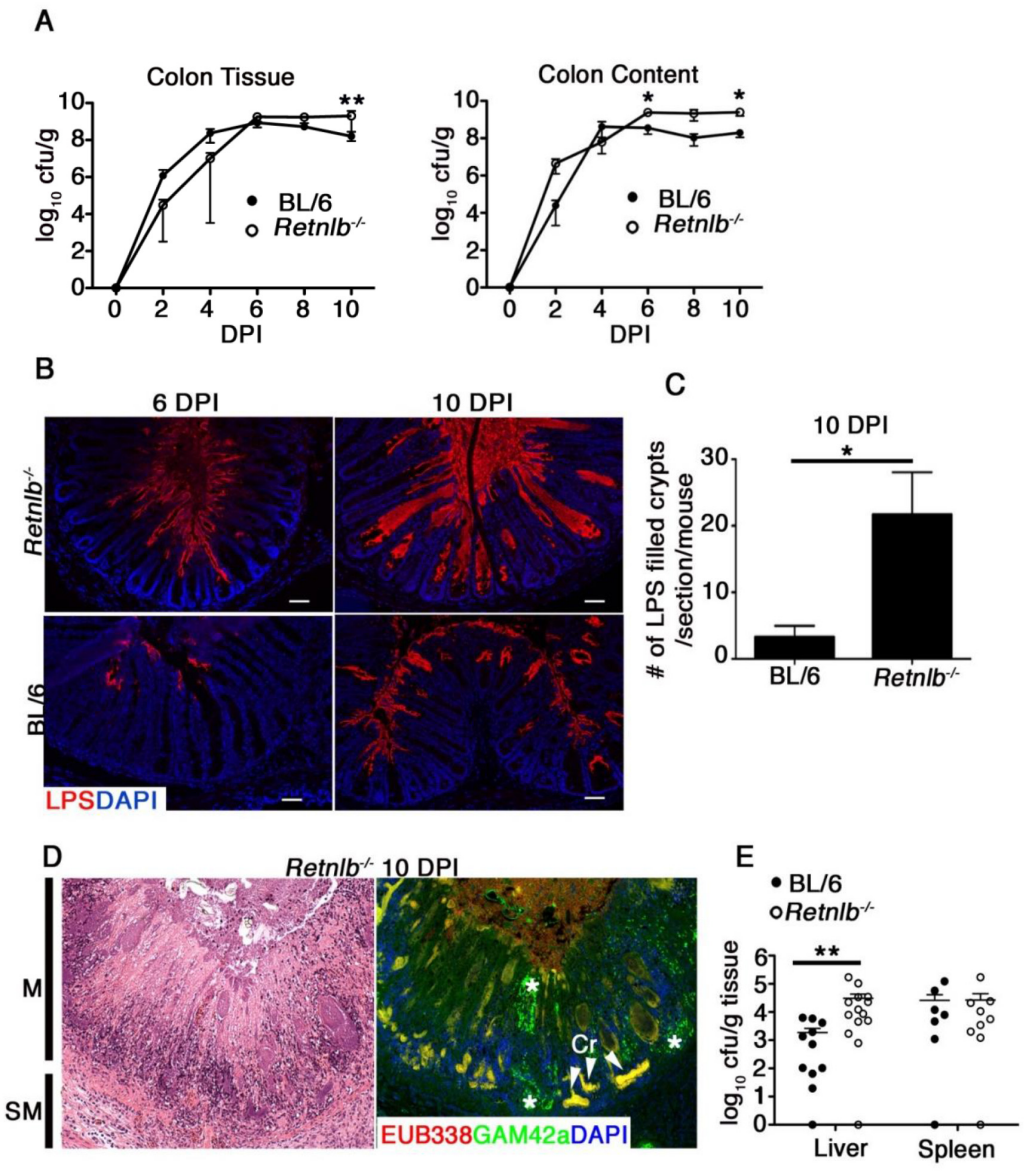
Disease severity of *Retnlb* ^*-/-*^ mice is associated with greater *C*. *rodentium* invasion of colonic crypts. (A) *C*. *rodentium* enumeration in the colon tissue and luminal compartments of *Retnlb* ^*-/-*^ and C57BL/6 following infection. Results show means of 3–4 (2 and 4 DPI) and 6–11 (6–10 DPI) animals, and are representative of 2 independent experiments **P = 0.0047, *P = 0.0262; ^##^ *P* = 0.0015 (*Retnlb* ^-/-^ vs. C57BL/6); Mann-Whitney *U*-test. (B) *C*. *rodentium* staining in mouse colonic tissues (6 & 10 DPI). Original magnification = 200X. Results are representative of 4–6 mice/group. (C) Quantitation of *C*. *rodentium* LPS-filled crypts in *Retnlb* ^*-/-*^ and C57BL/6 mice (10 DPI). Results represent mean of total crypts counted in single mice, pooled from 3–6 mice/group. *P < 0.05. (D) Left panel: H&E staining of heavily ulcerated colonic tissue from a *Retnlb* ^*-/-*^ mouse (10 DPI). M = Mucosa; SM = submucosa. Right Panel: dual FISH staining using a universal EUB338 probe (red) and Gamma-proteobacteria specific probe (green), revealing clusters of *C*. *rodentium* (yellow) deep within ulcerated tissue, presumably where crypt bases were located. *Cr—C*. *rodentium*. Results are representative of all ulcerated *Retnlb* ^*-/-*^ mice analyzed (n = 5). (E) Enumeration of systemic *C*. *rodentium* burdens in C57BL/6 vs. *Retnlb* ^*-/-*^ mice (10 DPI). Each data point represents one animal. **P = 0.0037, Mann-Whitney *U*-test.

To test whether the severe damage suffered by *Retnlb* ^-/-^ mice reflected heavier pathogen burdens, we quantified *C*. *rodentium* within the colons and ceca of *Retnlb* ^*-/-*^ and C57BL/6 mice up to 10 DPI. *C*. *rodentium* burdens adherent to the colonic and cecal tissues of *Retnlb* ^-/-^ mice were not significantly different than those recovered from C57BL/6 mice from 2 to 8 DPI, but were significantly (5 fold) higher at 10 DPI (Fig 3A and S2E Fig). Similarly, pathogen burdens found in the colonic and cecal stool contents of *Retnlb* ^-/-^ mice were modestly (but significantly) elevated over those burdens recovered from C57BL/6 mice at 6 and 10 DPI (Fig 3A and S2E Fig). Considering the exaggerated morbidity and pathology suffered by infected *Retnlb* ^-/-^ mice, the observed differences in overall pathogen burdens seemed relatively unimpressive, so we next examined whether *C*. *rodentium* localization differed between the mouse strains by staining tissues for *C*. *rodentium* LPS. Strikingly, *C*. *rodentium* was found to deeply penetrate the crypts of *Retnlb* ^-/-^ mice at 6 DPI, (Fig 3B). At 10 DPI, *C*. *rodentium* filled the lumens of many *Retnlb* ^-/-^ colonic and cecal crypts, from the surface epithelia down to crypt bases (Fig 3B and 3C and S2F Fig) likely representing the source of the modestly increased burdens we recovered from these mice. In contrast, *C*. *rodentium* predominantly localized to surface epithelia in C57BL/6 mice, and only rarely penetrated their crypts (Fig 3B and 3C and S2F Fig). This difference in crypt penetration and evidence that *C*. *rodentium* directly infected epithelial cells near the base of *Retnlb* ^-/-^ colonic crypts was confirmed by immunostaining for the bacterial T3SS effector Tir (S2G Fig). FISH staining of ulcerated colonic regions revealed clusters of exclusively *C*. *rodentium* (yellow) and not commensal bacteria (red) localized in patterns resembling crypts, suggesting ulcers formed at sites where *C*. *rodentium* had been deeply penetrating crypt bases (Fig 3D). Consistent with this deep tissue invasion and ulceration, significantly higher pathogen burdens (*P* < 0.005) were found in the livers of *Retnlb* ^*-/-*^ mice (Fig 3E). Interestingly, when we infected *Retnlb* ^*-/-*^ mice with a *C*. *rodentium* strain lacking EspF (*ΔespF*), a T3SS effector linked to IEC damage and barrier disruption, most disease parameters including weight loss and colonic ulceration were abrogated, showing that the exaggerated tissue damage that developed in infected *Retnlb* ^*-/-*^ mice required the full pathogenic properties of *C*. *rodentium* (S3A–S3D Fig).

### Infection-induced antimicrobial and inflammatory responses are intact in *Retnlb* ^-/-^ mice

The deep penetration of *C*. *rodentium* into *Retnlb* ^*-/-*^ mouse tissues led us to test whether RELM-β deficiency caused any defects in antimicrobial responses. Several antimicrobial genes (iNOS, mCRAMP, and RegIIIγ) primarily expressed by IEC were assessed. No defects in their expression were noted, and there was in fact a trend for elevated levels of RegIIIγ in the *Retnlb* ^*-/-*^ mice (S4A Fig). Moreover no defects were detected in the antimicrobial capacity of the colonic crypts themselves (S4B Fig). We also tested whether recombinant RELM-β itself possessed antimicrobial activity, and found that rRELM-β did not exhibit any *C*. *rodentium* killing capacity (S4C Fig).

We next examined whether inflammatory responses were impaired in the *Retnlb* ^-/-^ mice. Analysis of pro-inflammatory cytokine genes revealed heightened mRNA transcript levels for TNF-α, IL-1β, and IL-6 in the colons (S4D Fig) and ceca (S2B Fig) of infected *Retnlb* ^-/-^ vs C57BL/6 mice. We also found roughly similar numbers of macrophages and neutrophils infiltrating the colons of the two mouse strains by F4/80 and myeloperoxidase (MPO) staining respectively (S4E Fig), aside from areas of ulceration in the infected *Retnlb* ^-/-^ mice where neutrophils were found in much greater abundance, even by H&E staining (Fig 2D). Collectively, these studies indicate that loss of RELM-β does not cause any overt defects in launching antimicrobial or inflammatory responses to *C*. *rodentium* infection that could explain the deep crypt penetration seen in *Retnlb* ^-/-^ mice.

### *Retnlb* ^*-/-*^ mice exhibit reduced IEC proliferation during infection

**Fig 4:**
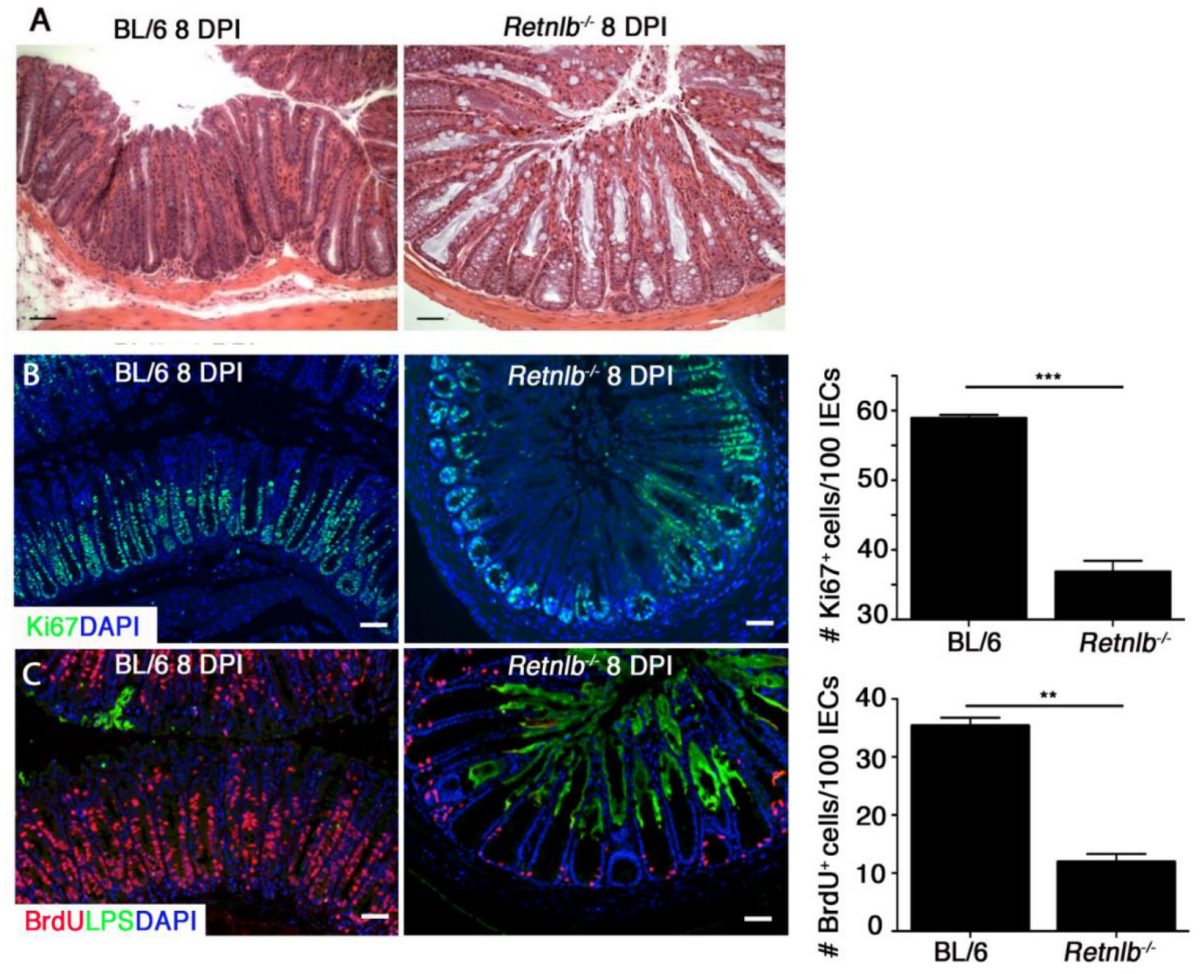
*Retnlb* ^*-/-*^ mice display defective epithelial proliferative responses predisposing to deep infection of colonic crypts. (A) H&E staining of infected colons at 8 DPI. Scale bar = 50 μm. Original magnification = 200X. (B) Ki67 staining (green) of infected colons (8 DPI) as well as DAPI staining of host cell nuclei (blue) as shown. Scale bar = 50 μm. Graph on right: Bars represent mean average value of Ki67^+^ cells/100 IECs, from 3 mice/group. (C) Dual labeling for BrdU (red) and LPS (green) in C57BL/6 and *Retnlb* ^*-/-*^ mice. Original magnification = 200X. Scale bar = 100 μm. Graph on right: Bars represent the mean average number of BrdU^+^ cells per 100 cells in 3 mice/group. Error bars = SEM. **P = 0.0068, ***P = 0.0002. Results are representative of at least 3 independent experiments.

While exploring whether other host defenses might be compromised in *Retnlb* ^*-/-*^ mice, we observed through H&E staining that their colonic crypt structures did not change during infection in the same manner seen in C57BL/6 mice. This was most notable at 8 and 10 DPI, when the *Retnlb* ^*-/-*^ crypts showed less cellularity, wider lumens, and more mature goblet cells than those in C57BL/6 mice (Fig 4A). Since *C*. *rodentium* infection is known to dramatically increase IEC proliferation, leading to mature goblet cell depletion [12, 13], we examined IEC proliferation in the two mouse strains by staining for the proliferation marker Ki-67 [36]. While no baseline differences were noted between strains (S5A and S5B Fig) (9.3 ± 0.4 versus 10.2 ± 0.7 Ki67 +ve cells/crypt); at 8 DPI we saw significantly increased numbers of Ki67^+^ IEC in the colons of C57BL/6 mice (30.8 ± 1.5) whereas this IEC hyper-proliferative response was significantly impaired (16.8 ± 1.7, *P <* 0.005) in the *Retnlb* ^*-/-*^ mice (Fig 4B). Over the next six days, IEC proliferation gradually increased such that by 14 DPI, the reduction in IEC proliferation in *Retnlb* ^*-/-*^ mice that survived to that point (versus C57BL/6 mice) was no longer observed (both > 70 +ve cells/ crypt) suggesting that while RELM-β was not essential for IEC proliferation, the absence of RELM-β caused a major delay in IEC proliferative responses to *C*. *rodentium* infection.

Based on this data, we sought to determine whether the induction of IEC hyper-proliferation was related to the ability of *C*. *rodentium* to deeply penetrate colonic crypts. We analyzed IEC proliferation using 5-bromodeoxyuridine (BrdU) incorporation, and performed dual labeling for BrdU and *C*. *rodentium* LPS. The immunostaining revealed *C*. *rodentium* colonization in C57BL/6 mice was limited to the surface epithelium of crypts displaying abundant BrdU^+^ IEC extending from the crypt base to the surface (Fig 4C). However, in *Retnlb* ^*-/-*^ mice, *C*. *rodentium* deeply invaded many of the colonic crypts showing only sparse numbers of BrdU^+^ IEC limited to the crypt bases (Fig 4C). Overall, consistent with Ki67 staining, BrdU labeling was significantly reduced (*P* < 0.01) by approximately 4-fold in *Retnlb* ^*-/-*^ mice vs. C57BL/6 mice at 8 DPI (Fig 4C). As shown in Fig 3B, the number of LPS-filled crypts was increased 5-fold in *Retnlb* ^*-/-*^ mice, suggesting that their impaired IEC proliferative responses coincided with their crypts being highly susceptible to deep *C*. *rodentium* penetration. Notably, we previously identified deep *C*. *rodentium* colonization of colonic crypts exhibiting limited IEC proliferation in *Rag1* ^*-/-*^ mice [13].

### CD4^+^ T cells are sufficient to induce IEC proliferative responses during infection

**Fig 5:**
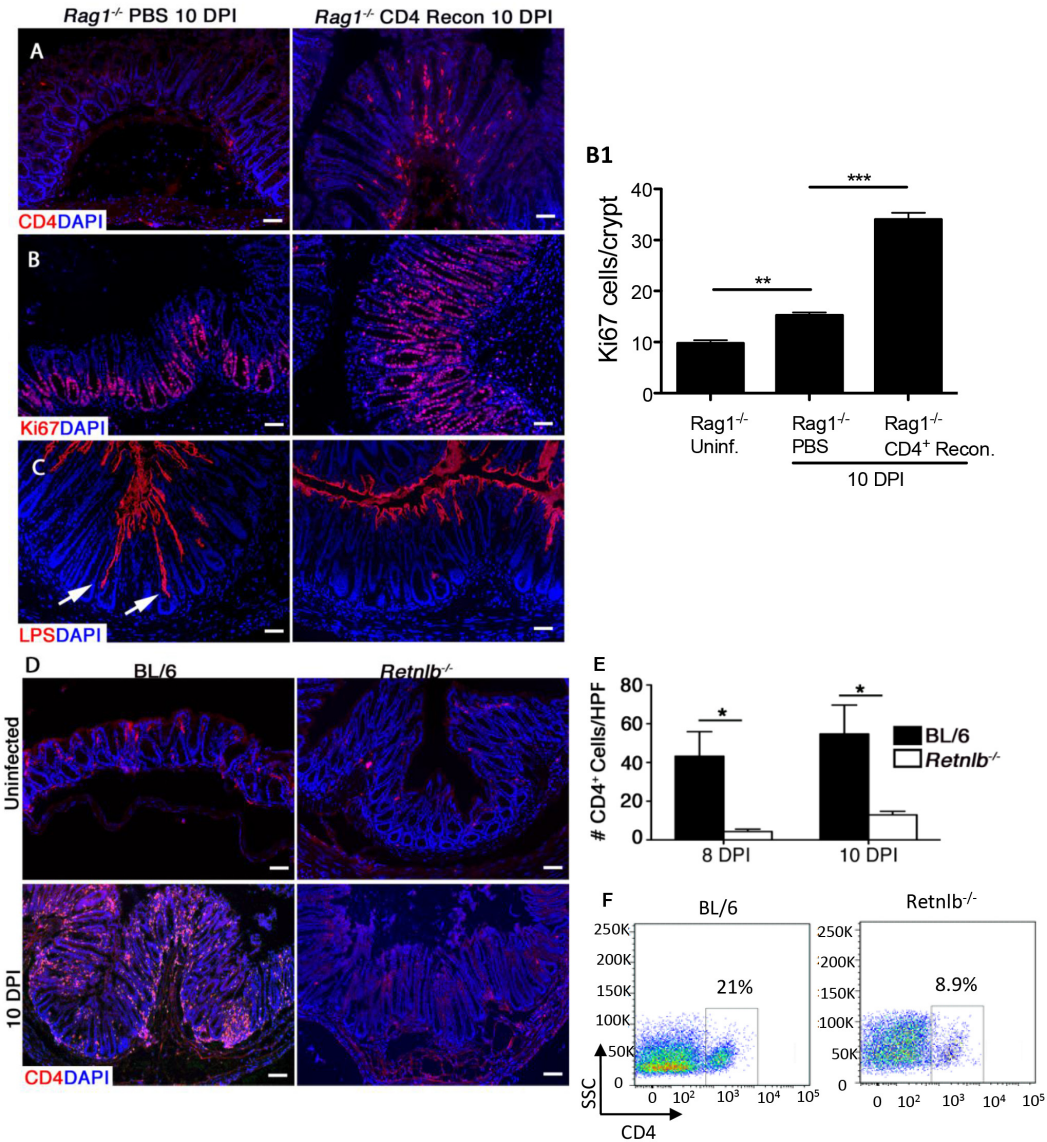
Reduced mucosal CD4^+^ T-cell numbers within infected *Rag1* ^*-/-*^ and *Retnlb* ^*-/-*^ mice, leading to defective epithelial cell proliferation. (A) CD4 staining in the colons of infected *Rag1* ^*-/-*^ mice reconstituted with either PBS or CD4^+^ T cells. (B) Ki67 staining in PBS or CD4^+^ T cell-reconstituted *Rag1* ^*-/-*^ mice (10 DPI). Graph on right (B1): Enumeration of Ki67-labeled cells. Bars represent mean average number of Ki67^+^ cells/100 cells. Error bars = SD. *P < 0.01. (C) LPS staining showing deeper penetration of *C*. *rodentium* into the crypts of PBS-reconstituted *Rag1* ^*-/-*^ mice (arrows) as compared to CD4^+^ T cell-reconstituted *Rag1* ^*-/-*^ mice (10 DPI). Scale bar = 100 μm. Original magnification = 200X. (D) CD4 staining in uninfected or 10 DPI C57BL/6 and *Retnlb* ^*-/-*^ mice. (E) Enumeration of CD4^+^ cells within colonic sections. Results represent means of cells counted from 5–10 high power fields (400X)/mouse. *P < 0.05. Error bars = SEM. (F) Flow cytometry of lamina propria lymphocytes purified from infected C57BL/6 and *Retnlb* ^*-/-*^ mice (8 DPI). All results are representative of 2–3 independent experiments, with 3–5 mice/group.

Crypt hyperplasia and increased IEC proliferation are common features of many infectious as well as non-infectious GI diseases, including tropical enteropathy and celiac disease [37, 38]. While the underlying mechanisms are unclear, T cell activation has been repeatedly linked to these pathologies. Moreover, we recently showed that adoptive transfer of CD4^+^ T cells into *Rag1* ^*-/-*^ mice increases IEC proliferation during *C*. *rodentium* infection [13]. Based on our finding of impaired IEC proliferation in *Retnlb* ^*-/-*^ mice, we examined whether CD4^+^ T cells might control IEC proliferation by modulating intestinal RELM-β levels. We therefore tested how reconstituting CD4^+^ T cells into *Rag1* ^*-/-*^ mice affected both IEC proliferation, as well as RELM-β expression. At 10 DPI, colon tissues were immunostained for CD4^+^ cells, proliferative responses (Ki67) and pathogen colonization (LPS), as well as RELM-β. As expected, CD4^+^ cells were virtually absent in non-reconstituted *Rag1* ^*-/-*^ mice, but were abundant in the mucosa of CD4^+^ T cell-reconstituted *Rag1* ^*-/-*^ mice (Fig 5A), confirming successful reconstitution. Analysis of IEC proliferation at 10 DPI found that infected CD4+ reconstituted *Rag1* ^*-/-*^ mice showed a dramatic increase in Ki67^+^ IEC compared to non-reconstituted *Rag1* ^*-/-*^ mice (Fig 5B and 5B1). Importantly, LPS staining revealed *C*. *rodentium* deeply penetrating the crypts of non-reconstituted *Rag1* ^*-/-*^ mice, whereas it was limited to the surface epithelium of CD4^+^ reconstituted *Rag1* ^*-/-*^ mice (Fig 5C). When tissues were stained for RELM-β, abundant positively staining goblet cells were noted in both the non-reconstituted *Rag1* ^*-/-*^ mice as well as the CD4^+-^reconstituted *Rag1* ^*-/-*^ mice (S5C Fig). These results indicate not only that induction of RELM-β expression does not require CD4^+^ T cells, but more importantly, that RELM-β is insufficient, in the absence of CD4^+^ T cells, to drive IEC hyper-proliferation during *C*. *rodentium* infection.

### Infected *Retnlb* ^*-/-*^ mice display reduced numbers of colonic CD4^+^ T cells

The above results suggested that the impaired IEC proliferation seen in *Retnlb* ^*-/-*^ mice might reflect some defect in their CD4^+^ T cells, however baseline CD4^+^ T cell numbers within the intestine and spleen are known to be similar in *Retnlb* ^*-/-*^ vs C57BL/6 mice [24, 33]. Alternatively, we hypothesized that *Retnlb* ^*-/-*^ mice might suffer an impaired ability to recruit CD4^+^ T cells to the colon upon infection, since an inability to recruit these cells could potentially explain their impaired IEC proliferation during infection. We therefore immunostained colonic tissues from uninfected and infected C57BL/6 and *Retnlb* ^*-/-*^ mice, for the marker CD4. While CD4^+^ cells were sparse in both strains under uninfected conditions (Fig 5D), at 10 DPI, numerous intensely stained CD4^+^ cells with distinct lymphocyte morphology were seen in the submucosa and mucosa of C57BL/6 mice (Fig 5D). In contrast, CD4^+^ cell numbers were significantly reduced (*P* < 0.05) in *Retnlb* ^*-/-*^ mice, at roughly 15–25% the number found in C57BL/6 colons at both 8 and 10 DPI (Fig 5D and 5E). These results were confirmed by FACs analysis of cells isolated from the colonic lamina propria from the two mouse strains (Fig 5F). Furthermore, FACs analysis of CD8+ve T cell populations showed no significant differences between C57BL/6 and *Retnlb* ^*-/-*^ mice, indicating that loss of RELM-β did not affect all T cell populations. These data suggest that the paucity of CD4^+^ T cells in the mucosa of *Retnlb* ^*-/-*^ mice is likely the basis for their stunted IEC proliferative responses to *C*. *rodentium* infection.

### Adaptive immune responses to *C*. *rodentium* are not compromised in *Retnlb* ^*-/-*^ mice

While CD4^+^ T cells are sufficient to drive increased IEC proliferation, it was unclear whether the reduced CD4^+^ T cell numbers found in infected *Retnlb* ^*-/-*^ mice reflected a defect in their priming, or in their recruitment to the site of infection. However, stimulation with *C*. *rodentium* antigen revealed similar antigen-specific secretion of IL-17A and IFNγ from splenocytes isolated from infected C57BL/6 and *Retnlb* ^*-/-*^ mice (S6A and S6B Fig). These results suggest that priming of adaptive immune responses to *C*. *rodentium* is not compromised in the absence of RELM-β.

### RELM-β exhibits direct chemotactic activity for CD4^+^ T-cells *in vitro*

**Fig 6:**
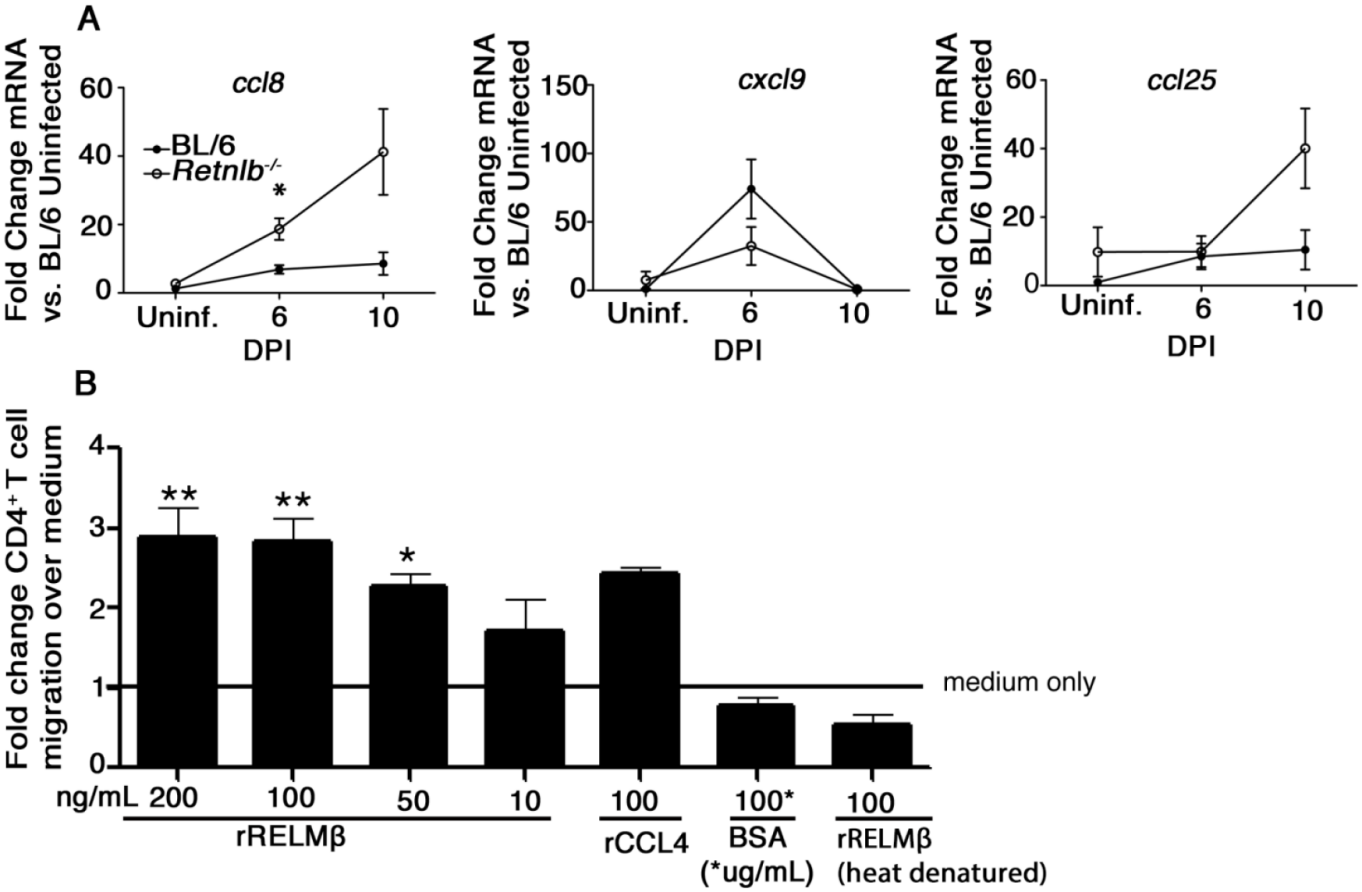
RELM-β acts as a CD4+ T cell chemoattractant. (A) qPCR analysis of chemokine genes involved in T-cell recruitment in colonic tissues of uninfected or infected C57BL/6 and *Retnlb* ^*-/-*^ mice at 6 and 10 DPI. Results are means of n = 4–8 mice/group, pooled from 2 independent experiments. *P < 0.05 *Retnlb* ^*-/-*^ 6 DPI vs. C57BL/6 UI, Bonferroni’s post-test following 1-way ANOVA. (B) Graph showing percent CD4^+^ T cells migrating toward RELM-β in a chemotaxis assay. Results are representative of 3 independent experiments, 4 replicates/group. *P < 0.05; **P < 0.001. Error bars = SEM.

We next tested whether *Retnlb* ^*-/-*^ mice suffered defects in recruiting CD4^+^ T-cells to the colon, by measuring colonic gene expression of the T cell chemokines CCL8, CXCL9, and CCL25 [39]. We found significantly enhanced gene expression of CCL8 at 6 DPI, while both CCL8 and CCL25 were increased (but not significantly) at 10 DPI in *Retnlb* ^*-/-*^ mice, compared to that found in infected C57BL/6 mice (Fig 6A). While CXCL9 expression was mildly impaired at 6 DPI, it was not significant, and did not seem at a level that would have such a major effect on CD4^+^ T-cell recruitment. With no overt defects identified in these key chemokine genes, we noted previous studies showing that RELM-β could directly chemoattract stromal cells to sites of lung injury [40]. We therefore examined whether RELM-β could itself recruit CD4^+^ T cells using a Boyden Chamber assay. Interestingly, rRELM-β caused a dose-dependent increase in CD4^+^ T cell migration, similar to that seen using the T-cell chemokine CCL4 (Fig 6B), but the chemoattraction was abrogated when rRELM-β was heat inactivated. Therefore the impaired T cell recruitment seen in *Retnlb* ^*-/-*^ mice likely reflects the loss of RELM-β and its ability to chemoattract CD4^+^ T cells to the infected colon.

### IL-22 increases IEC proliferation and is significantly reduced in *Retnlb* ^*-/-*^ mice

**Fig 7:**
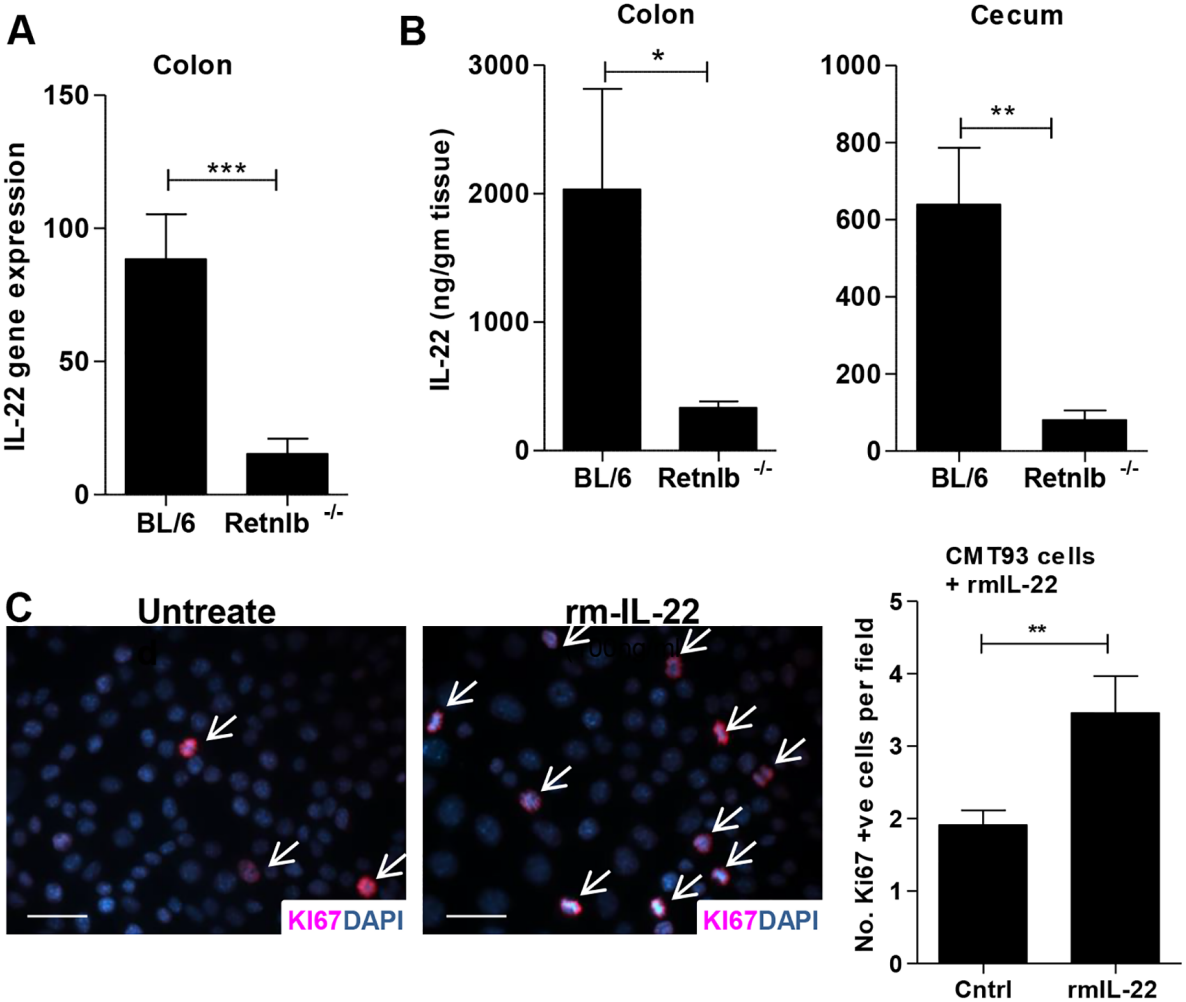
IL-22 induces IEC proliferation *in vitro* and its production is impaired in *Retnlb* ^*-/-*^ mice. (A) qPCR analysis of IL-22 gene transcripts in colonic tissues of infected C57BL/6 and *Retnlb* ^*-/-*^ mice at 8 DPI. (B) IL-22 protein levels measured via ELISA in colonic (left) and cecal (right) tissues of infected C57BL/6 and *Retnlb* ^*-/-*^ mice at 8 DPI. Results are means of n = 6 mice/group, *P < 0.05, **P < 0.005, ***P < 0.0005. All error bars = SEM. (C) Ki67 staining and enumeration of Ki-67+ CMT93 cells untreated (PBS) or treated with recombinant mouse IL-22 in culture. Bars represent average number of Ki67+ cells per 60X field. Fig 7A and 7B show results representative of 3 independent experiments with n = 4–10 mice per group and Fig 7C was presented as an average of 3 independent experiments performed with at least 3 replicates per condition. **P < 0.01.

To determine the mechanism through which CD4+ T cells drive IEC proliferation during infection, we next examined levels of IL-22; a cytokine produced by CD4+ T cells during enteric infection that interacts with its receptors expressed by IEC [41]. IL-22 is unique amongst the interleukins in that its receptors are exclusively expressed on tissue resident non-hematopoietic cells such as IEC, inducing proliferative and antimicrobial responses in these cells [9, 41]. In fact, in separate studies, we found that neutralizing IL-22 in mice during *C*. *rodentium* infection caused a major impairment in IEC proliferation. C57BL/6 mice treated with an anti-IL-22 antibody showed less colonic IEC proliferation (19.1±1.7 Ki67+ve IEC/crypt) than infected mice receiving PBS alone (30.4 ±1.7) (P = 0.009) or mice receiving control antibodies (32.0 ±4.3) (P = 0.058). Interestingly, gene transcript and protein analysis revealed significantly impaired (*P <* 0.05) induction of IL-22 in *Retnlb* ^*-/-*^ mice during infection as compared to C57BL/6 mice (Fig 7A and 7B). Further, *in vitro* application of rIL-22 to the CMT93 colonic epithelial cell line significantly increased (*P <* 0.005) proliferation (Ki67+ cells) (Fig 7C), confirming the potential for its direct proliferative effects on IECs. In contrast, direct application of rRELM-β to this IEC line did not significantly increase Ki67+ cell numbers (S7A Fig). Since IL-22 can be produced by several cell types, including T lymphocytes and innate lymphoid cells, we compared its expression during infection in mice lacking T cells (*Tcr-β* ^*-/-*^) and C57BL/6 mice. We found that *Il-22* gene transcription, as well as IL-22 protein levels were dramatically reduced in the colons of *Tcr-β* ^*-/-*^ mice indicating that CD4^+^ T cells are the primary source of IL-22 at this time point in the infection (S7B and S7C Fig). These results thus suggest that IL-22 production by CD4^+^ T cells recruited to the infected intestine in response to RELM-β drives the protective IEC proliferative responses seen during infection.

### RELM-β enemas restore CD4^+^ T cells, IL-22 levels and IEC proliferation in infected *Retnlb* ^*-/-*^ mice

**Fig 8:**
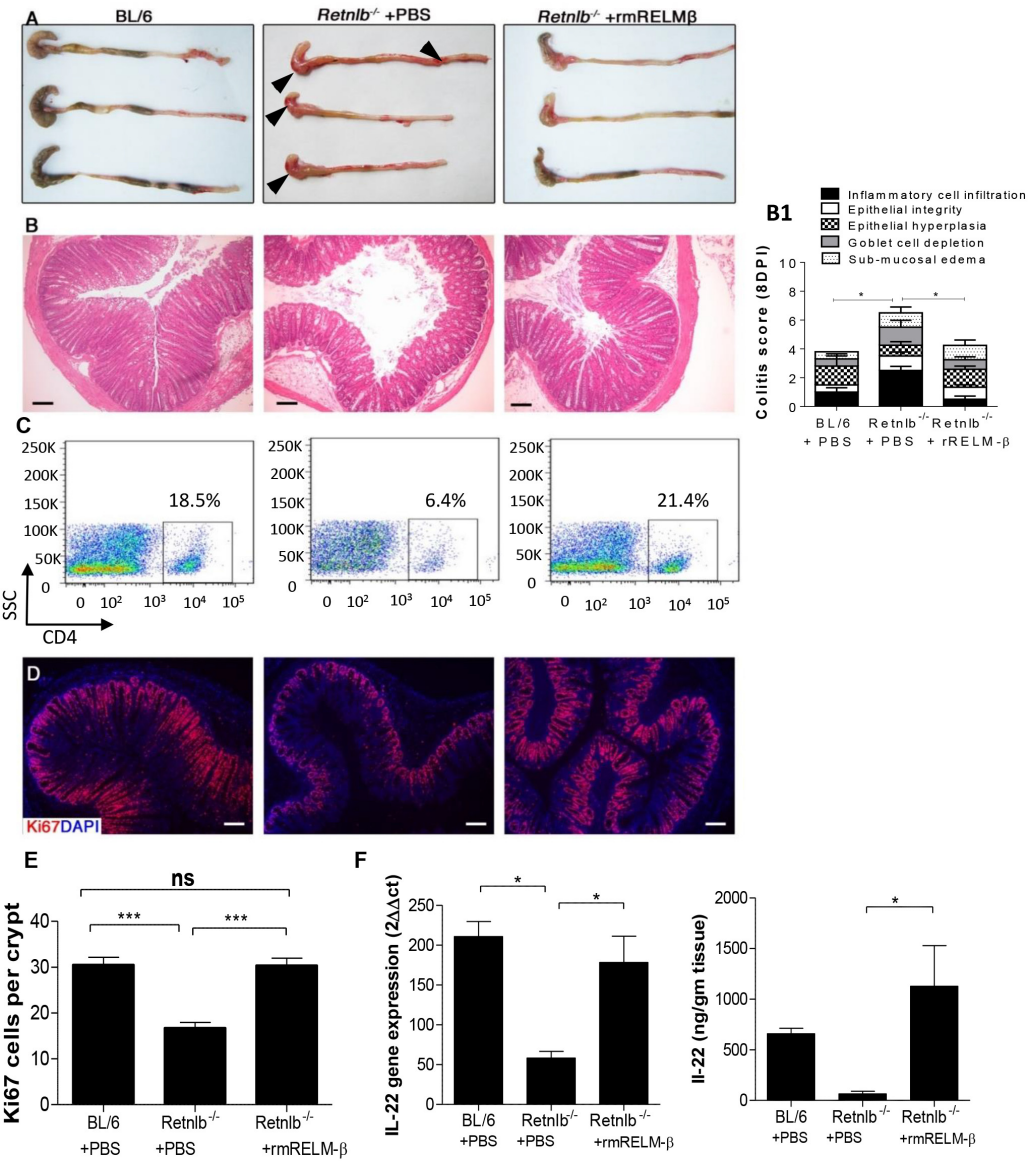
Reconstitution with recombinant RELM-β protects infected *Retnlb* ^*-/-*^ mice. (A) Resected colons + ceca from C57BL/6 mice or *Retnlb* ^*-/-*^ mice treated with either rRELM-β protein (400 ng) or vehicle (8 DPI). (B) H&E staining of colon tissues at 8 DPI (left panel) and their histopathological scoring (right panel B1) in the above mentioned mice. Scale bar = 100 μm. (C) Flow cytometry of CD4^+^ cells isolated from infected colons (8 DPI). (D) Colonic Ki67 staining (8DPI) in representative mice of each group, with enumeration (E) on right as in Fig 5E. (F) IL-22 gene transcript (left) and protein (right) levels measured via qPCR and ELISA, respectively, in infected C57BL/6 and *Retnlb* ^*-/-*^ mice (treated with PBS or rRELM-β protein). *P < 0.05; ***P < 0.0001. Error bars = SEM. Scale bar = 100 μm. Fig 8A, 8B, 8B1 and 8D are representative of 4 independent experiments with n = 3 mice/group. The pathology scoring represents an average of these experiments.

To clarify whether restoring RELM-β could protect *Retnlb* ^*-/-*^ mice during infection, the effects of delivering rRELM-β into *Retnlb* ^*-/-*^ mice were tested. Repeated intraperitoneal (i.p) injection of murine rRELM-β had no protective effect on infected *Retnlb* ^*-/-*^ mice (S8 Fig). We next tried enema delivery, with infected *Retnlb* ^*-/-*^ mice given 400 ng doses of rRELM-β in 1% methyl propyl cellulose in PBS, or the vehicle alone (control), by enema each day between 5 to 7 DPI, and the mice were euthanized at 8 DPI. Once euthanized, the protective effects of rRELM-β were readily apparent as the macroscopic intestinal injury and bleeding seen in the *Retnlb* ^*-/-*^ mice was dramatically attenuated following rRELM-β treatment (Fig 8A). Moreover the colonic pathological scores as well as levels of crypt hyperplasia in the *Retnlb* ^*-/-*^ mice given rRELM-β enemas were found to be similar to that seen in C57BL/6 mice, and significantly different from those seen in vehicle treated *Retnlb* ^*-/-*^ mice (Fig 8B and 8B1). As assessed by FACs analysis, rRELM-β treatment led to a marked increase in CD4^+^ T cell numbers populating the colonic mucosa (Fig 8C). Significantly increased numbers of Ki67^+^ proliferating IEC (*P <* 0.0001) were also observed in rRELM-β treated mice (Fig 8D and 8E). Moreover significantly increased IL-22 production (*P <* 0.05) was observed at both gene transcript and protein levels in rRELM-β treated mice (Fig 8F). These results thus indicate that luminal delivery of rRELM-β to *Retnlb* ^*-/-*^ mice recruits CD4^+^ T cells to the infected colon; protecting the host by boosting IL-22 levels as well as proliferative IEC responses to replace infected and damaged cells.

## Discussion

Intestinal goblet cells are considered important contributors to intestinal barrier function, through the release of the mucin Muc2 and their critical role in the generation of the intestinal mucus layer. While production of intestinal mucus is generally considered a passive means of host defense, herein we report that goblet cells can also play an active and critical role in driving inflammation and intestinal remodeling, through the production of RELM-β. In contrast to other goblet cell mediators (Muc2 and TFF3), RELM-β expression was strongly induced during *C*. *rodentium* infection. Moreover RELM-β production proved protective, since infected *Retnlb* ^*-/-*^ mice suffered exaggerated mucosal damage compared to C57BL/6 mice. Loss of RELM-β resulted in deeper pathogen penetration of colonic crypts, patchy ulceration, as well as heightened morbidity and mortality. The protective actions of RELM-β did not appear to involve modulation of antimicrobial defenses, but rather reflected its role in recruiting CD4^+^ T cells to the infected colon, where they drove a protective increase in IEC proliferation through the production of IL-22.

Our previous studies demonstrated that goblet cell-derived Muc2 protected mice during *C*. *rodentium* infection, by limiting the pathogen’s ability to reach the underlying IEC [23]. However once pathogens successfully cross the intestinal mucus layer, the protective actions of Muc2 are overshadowed by the host’s immune response. Thus a major impetus for the current study was to examine whether goblet cells respond to a successful infection by changing the mediators they release. We focused our attention on RELM-β since it is strongly induced in several models of colitis, yet its function is poorly defined. In fact, whether RELM-β plays a pro- or anti-inflammatory role during colitis remains controversial. Reports have shown that RELM-β aggravates pathology during DSS-induced colitis, by activating macrophages and by increasing their production of TNF-α [32]. Another study suggested that RELM-β promotes the chronicity of *T*. *muris* infections as well as the severity of colitis by increasing IFNγ production by CD4^+^ T cells [33]. In contrast, in the TNBS colitis mouse model, RELM-β deficiency was shown to worsen disease via unknown mechanisms and correspondingly, enema delivery of rRELM-β was shown to ameliorate TNBS colitis [42]. Collectively, these results indicate the biological function of RELM-β may be context dependent, while our findings suggest its impact may reflect whether recruitment of CD4^+^ T cells to the intestine provides protection within the specific model tested, or alternatively, simply worsens colitis.

In our model, we noted a rapid increase in RELM-β expression by intestinal goblet cells in response to *C*. *rodentium* infection. While RELM-β gene transcript levels remained elevated throughout the course of infection, RELM-β protein levels peaked at 6 DPI and declined by 10 to 14 DPI, in keeping with the increasingly immature state of goblet cells at these later time points [12]. Aside from goblet cells being strongly immunoreactive for RELM-β, the protein was also detected in large quantities within the stool. This suggests RELM-β is released apically into the intestinal lumen, however consistent with a role for RELM-β in recruiting T cells to the infected intestine, it was also found elevated within the sera of infected mice. At present it is unclear whether this reflects basolateral release of RELM-β by the goblet cells, or instead leakage of the luminally secreted molecule across a disrupted epithelial barrier. Recent studies have shown that goblet cells can act as a passageway for luminal antigens to cross the epithelium and reach underlying immune cells [43]. Presumably such a role would also facilitate the passage of luminal RELM-β into the underlying lamina propria and beyond.

Consistent with its strong induction in our model, RELM-β played an important protective role, as mice lacking the protein suffered more severe colitis than WT mice. The exaggerated tissue damage reflected the deep penetration of *Retnlb* ^*-/-*^ crypts by *C*. *rodentium*, resulting in direct pathogen damage to the epithelium as well as host immune-mediated loss of crypts and patchy ulceration. Considering that *C*. *rodentium* typically colonizes IEC at the surface of crypts, its presence at the crypt bases suggested the *Retnlb* ^*-/-*^ mice were impaired in host defense. While no overt defects in antimicrobial or inflammatory responses were detected, the *Retnlb* ^*-/-*^ mice did suffer a striking delay in their ability to induce the IEC hyper-proliferation typically seen during *C*. *rodentium* infection. We and others have shown this process increases the turnover and replacement of infected IEC and is driven by the host’s immune system [12–13, 44]. This process, previously described as an “epithelial escalator” has been highlighted as a protective response during *Trichuris muris* infection [45], and our data suggests this process also benefits the host during infection by a mucosal adherent bacterial pathogen.

While increasing IEC proliferation and ultimately sloughing IEC into the intestinal lumen may help keep mucosal adherent pathogens away from the base of intestinal crypts, an equally important protective role may be the replacement of damaged epithelial cells, as a form of regeneration and restitution of the epithelial layer. We propose this is a key aspect of host defense against *C*. *rodentium* infection, promoting tissue tolerance (the ability of the host to survive the infection), by limiting the extent of damage suffered during infection. While tissue tolerance has been primarily highlighted as a strategy by plants to survive infection [46], recent studies have begun to address its relevance in mammalian hosts [17]. We have also shown that TLR2 mediated maintenance of IEC barrier function during *C*. *rodentium* infection plays a critical role in limiting tissue damage and mortality, without impacting on pathogen burdens [18]. This suggests that promoting tissue tolerance is an important goal of the host immune system in this model. Notably, while increased IEC proliferation may be a requirement for host survival during *C*. *rodentium* infection, it is not on its own sufficient to overcome severe genetic susceptibilities in other aspects of host defense. For example, mice deficient in TNF-α as well as *Muc2* ^*-/-*^ mice exhibit significant susceptibility to *C*. *rodentium* infection, despite undergoing IEC hyper-proliferation. Thus IEC hyper-proliferation appears to work with other mechanisms of host defense to provide optimal protection against *C*. *rodentium* infection.

RELM-β’s role in driving IEC proliferation appears to be indirect, as exposing IEC to RELM-β in culture did not lead to increased proliferation. Moreover, although RELM-β is strongly induced in infected *Rag1* ^*-/-*^ mice, these mice develop only modest increases in IEC proliferation in response to *C*. *rodentium*. Instead RELM-β appears to require an intact immune system, and specifically CD4^+^ T cells to drive crypt IEC proliferation and hyperplasia. *Retnlb* ^*-/-*^ mice showed a selective reduction in CD4^+^ T cell numbers within the large bowel during infection, as compared to wildtype mice. We determined that RELM-β itself has direct chemoattractive activity for CD4^+^ T cells. While previous studies have shown that RELM-β can modulate cytokine production by CD4^+^ T cells [33], this is the first study to show it recruits these cells to sites of injury/infection. While there are several other chemokines known to recruit CD4^+^ T cells, a previous study showed that reduced production of IEC-derived chemokines (such as CCL25) during *C*. *rodentium* infection had little impact on CD4^+^ T cell recruitment at early stages (7 DPI) but only impacted CD4^+^ T cell numbers at later stages of infection (14 DPI) [47]. Why traditional chemokines have such a delayed effect in this model is unclear, however this delay in their actions may explain why the very rapid upregulation and release of RELM-β plays such an important role in protecting the host during the early stages of *C*. *rodentium* infection.

While CD4^+^ T cells can produce a number of cytokines, depending on their functional phenotype, *C*. *rodentium* infection is noted for the localized recruitment of Th22 CD4^+^ T cells to the colon, where they produce large quantities of the cytokine IL-22. A multifunctional cytokine, IL-22 has been shown to induce antimicrobial responses as well as promote cellular proliferation [9, 41], and we confirmed that IL-22 directly increases IEC proliferation in culture. While several studies have implicated innate lymphoid cells (ILC) as important producers of IL-22 during *C*. *rodentium* infection [48], the fact that *Rag1* ^*-/-*^ mice possess ILC in their intestines, but develop little increase in IEC proliferation during infection suggests ILC are not primarily involved in the IEC hyper-proliferative response. This may reflect the overall levels of IL-22 produced by ILC versus Th22 cells, or alternatively the localization of these cells during the course of infection. Similarly, while IL-22 has been shown to be required for the full induction of antimicrobial responses during *C*. *rodentium* infection [41], we noted no overt defect in antimicrobial defenses in *Retnlb* ^*-/-*^ mice, suggesting that the levels of IL-22 found in their tissues are sufficient to drive these responses, but insufficient to promote IEC proliferation.

RELM-β’s ability to drive CD4^+^ T cell recruitment to the gut may in part explain its complicated effects during parasite infections [25, 33–34], as well as its worsening effect on several models of colitis [31, 32]. Interestingly, RELM-β is not the only RELM family member to play a role during *C*. *rodentium* infection. RELM-α is produced primarily by macrophages, and was recently shown to promote a Th17 immune response by priming naive T-cells through increased MHCII expression and by increasing IL-23 expression by macrophages [49]. In contrast, we did not observe overt defects in T cell priming in the absence of RELM-β, suggesting distinct non-redundant functions of specific RELM family members in immunity to *C*. *rodentium* infection. Overall, our findings expand the role attributed to goblet cells in providing host defense against enteric bacteria. While intestinal mucus provides an important barrier against intestinal microbes, once that barrier is bypassed, the host’s immune system must be recruited to deal with invading pathogens. The induction of RELM-β within goblet cells, and its subsequent release may reflect a generalized but effective host approach to rapidly recruit CD4^+^ T cells to the intestine to deal with noxious stimuli threatening intestinal integrity. Considering the array of colitic stimuli shown to upregulate RELM-β expression, it will be interesting to define whether RELM-β promotes T cell recruitment in other models of colitis.

## Material and Methods

### Mice

Six to twelve-week-old *Retnlb* ^*-/-*^ and *Tcr-β* ^*-/-*^ mice (both generated on a C57BL/6 genetic background) and wildtype (C57BL/6) mice were bred in the animal facilities at the Child and Family Research Institute (CFRI), whereas *Rag1* ^*-/-*^ mice were purchased from The Jackson Laboratory. Mice were kept in sterilized, filter-topped cages, handled in tissue culture hoods and fed autoclaved food and water under specific pathogen free (SPF) conditions. Sentinel animals were routinely tested for common pathogens.

### Ethics statement

All experiments were performed according to a protocol (A11-0290) approved by the University of British Columbia's Animal Care Committee and in direct accordance with The Canadian Council on Animal Care (CCAC) guidelines. Mice were monitored for mortality and morbidity throughout their infection and euthanized if they showed signs of extreme distress or >15% body weight loss.

### Bacterial strains, infection and rRELM-β injections and enemas

Mice were infected by oral gavage with 0.1 ml of an overnight culture of Luria-Bertani (LB) broth grown at 37°C with shaking (200 rpm) containing 2.5 x 10^8^ cfu of *C*. *rodentium* (strain DBS100) [23, 50]. For natural infection studies, the protocol was modified from Wiles *et al*. [51]; briefly, C57BL/6 mice were infected by oral gavage as described above. After colonization was confirmed by plating of stool contents (described below), a single mouse harboring over 10^8^ cfu/g of stool was placed in a cage of uninfected BL/6 mice (n = 3–4). Infected and non-infected mice remained co-housed, and stool was taken at 4 and 7 PI to determine colonization. For RELM-β injections, 10μg recombinant (r)Relmβ in sterile PBS (100μL of 100μg/mL), or PBS alone as vehicle control, was administered intraperitoneally to *Retnlb* ^*-/-*^ mice 2 days prior to infection and every second day following infection. Mice were euthanized at 10 DPI and analyzed as above. For RELM-β enemas, mice were injected with 200μl of 2μg/ml rRELM-β in 1% methyl propyl cellulose in phospho-buffered saline (PBS), while control mice received just 1% methyl propyl cellulose in PBS. At 5 days post-infection (DPI), two doses of RELM-β were given, followed by single doses at 6 and 7 DPI. Mice were euthanized at 8 DPI to carry out tissue analysis.

### Tissue collection

Tissues for histology, mRNA and cryosectioning were prepared as previously described [23, 50]. In brief, mice were anesthetized with halothane, euthanized by cervical dislocation, and their large bowel resected and regionally divided. Cecal and distal colonic tissues were immediately placed in 10% neutral buffered formalin (Fisher) (48 hrs, 4°C) or ice cold fresh Carnoy’s Fixative (2 hrs, 4°C) or 4% paraformaldehyde (PFA) (1 hr, room temp) for histological studies, or placed in RNA*later* (Qiagen) and stored at -86°C for subsequent RNA and protein extraction. For histology, tissues collected in formalin or Carnoy’s were transferred into 70% or 100% ethanol, respectively (after they underwent the appropriate fixation time), embedded in paraffin and cut into 5-μm sections. PFA fixed tissues were embedded in Optimal Cutting Temperature (OCT) compound and sectioned with a microtome-cryostat.

### Bacterial counts

For enumeration of *C*. *rodentium* within intestinal tissues and luminal compartments, whole mouse colons and ceca were opened longitudinally, and their luminal contents collected in pre-weighed 2.0 ml microtubes containing 1.0 ml of PBS and a 5.0 mm steel bead (Qiagen). Intestinal tissues were washed vigorously in PBS (pH 7.4), cut into several pieces, and also placed in a tube as above. Tissue and lumen contents were weighed, and then homogenized in a MixerMill 301 bead miller (Retche) for a total of 6 mins at 30 Hz at room temperature. Tissue homogenates were serially diluted in PBS and plated onto luria broth (LB) agar plates containing 100 mg/ml streptomycin, incubated overnight at 37°C, and bacterial colonies were enumerated the following day, normalizing them to the tissue or stool weight (per gram). A similar method was used to enumerate *C*. *rodentium* within the spleens and livers of infected mice.

For fecal bacterial burden analysis, stool was collected from live mice at various times post-infection (described in text) and processed as described for luminal contents. For some studies with non-antibiotic resistant *C*. *rodentium*, plating was performed on MacConkey Agar (Difco), *C*. *rodentium* colonies were clearly identified by their unique characteristic of being round with red centre and a thin white rim. Colonies were confirmed to be *C*. *rodentium* by PCR for the *C*. *rodentium* T3SS translocator gene *escN*.

### Histology scoring and immunofluorescence staining

Preparation of paraffin embedded slides for histological analysis (H&E) and immunostaining was carried out as previously described [9, 21]. H&E stained tissues were assessed for the following parameters by two blinded observers to determine extent of histological damage: (i) submucosal edema (0, no change; 1, mild; 2, moderate; 3, severe); (ii) goblet cell depletion (0, no change; 1, mild depletion; 2, severe depletion; 3, absence of goblet cells); (iii) hyperplasia (0, no change; 1, 1 to 50%; 2, 51 to 100%; 3, >100%); (iv) epithelial integrity (0, no pathological changes detectable; 1, epithelial desquamation [a few cells sloughed, surface rippled]; 2, erosion of epithelial surface [epithelial surface rippled, damaged]; 3, epithelial surface severely disrupted/damaged, large amounts of cell sloughing; 4, ulceration [with an additional score of 1 added for each 25% of tissue in the cross-section affected, e.g., a large ulcer affecting 70% of the tissue section would be scored 4 + 3]); (v) inflammatory cell infiltration (per ×400 magnification field) (0, no change; 1, <20; 2, 20 to 50; 3, >50 cells/field); the maximum possible pathology score using this scheme was 20. When ulcers were specifically assayed (see Fig 2E), small ulcers were defined as < 25% of tissue ulcerated in the cross-section; medium ulcers involved >25 to <50% of the cross section and large extensive ulcers involved >50% of the cross section.

For immunofluorescence staining 5-μm sections were deparaffinized by heating to 60°C for 15 min, cleared with xylene, rehydrated through an ethanol gradient to water, steamed for 30 min in citrate buffer for antigen retrieval, and blocked using blocking buffer (goat or donkey serum in PBS containing 1% bovine serum albumin [BSA], 0.1% Triton X-100, 0.05% Tween 20, and 0.05% sodium azide). The rabbit derived primary antibodies- RELM-β (Peprotech), MPO (Thermo Scientific), LPS (Biotec) and Ki67 (Thermo Scientific) were used at dilutions 1:400, 1:200, 1:500 and 1:200 respectively. Rat anti-murine antibodies F4/80 (AbD Serotec), CD4 (e-Bioscience), BrdU (AbD Serotec) and Tir (gift of Dr. Wanyin Deng) were used at dilutions 1:200, 1:100, 1:200 and 1:2000 respectively. Secondary goat anti-rabbit and anti-rat antibodies conjugated to AlexaFluor 488 and 568 were used at 1:2000 dilution and ProLong gold antifade reagent with 4′,6-diamidino-2-phenylindole (DAPI; Invitrogen) to stain DNA was used to mount tissues. Tissues were viewed on a Zeiss Axio Imager microscope, and images were taken using AxioVision software and an AxioCam HRm camera.

### RNA extraction and quantitative RT-PCR

Total RNA was extracted using an RNeasy kit (Qiagen) according to manufacturer’s instructions and quantified using a NanoDrop spectrophotometer. cDNA was synthesized using 1 μg of RNA reverse transcribed using an Omniscript RT kit (Qiagen). Quantitative PCR (qPCR) was performed on reactions containing 5 μL of 1:5 diluted cDNA in 10 μL of BioRad SYBR green mix with primers (300 nM final concentration) using an Opticon 2 (Bio-Rad) machine, as described previously [9]. Quantitation of data was carried out using Gene Expression Macro OM 3.0 software (Bio-Rad). Primer sequences and reaction conditions for all genes analyzed are given in S1 Table.

### Colonic crypt isolation and antimicrobial assays

Colon tissue was harvested and the distal 3 cm was washed 3x in sterile cold PBS, cut into 0.5 mm pieces and placed in a 2 ml microtube with1 ml crypt isolation buffer (30 mM EDTA, 2 mM DTT in PBS, pH 7.2), and gently rocked at 4°C for 20 minutes. The supernatant was collected, stored on ice and fresh isolation buffer was added to the tissues. This was repeated until 4 separate fractions were collected. On collection of the 4^th^ fraction, tissues were pulsed for 5s in a vortexor. The fractions were then centrifuged at 2000 rpm for 10 mins, 4°C, washed in 1 x PBS, pelleted again as above, the supernatant aspirated off, and samples were lysed in lysis buffer (1 x PBS + 0.1% TritonX-100 with 1x complete protease inhibitors (Roche)) and quantified. For the antimicrobial assay, an overnight culture of nalidixic acid-resistant *C*. *rodentium* grown in LB (+ nalidixic acid) was diluted 1:1000 in Tryptic Soy Broth (TSB) and grown to mid log phase (OD_620_ 0.6–1.0). The bacteria was washed by centrifugation (3000 rpm, 4°C, 10 mins), the supernatant removed, and the pellet resuspended in ice cold 10mM sodium phosphate buffer (SPB) (pH 7.4). This step was repeated once. The washed sample was diluted to a final OD_620_ of 0.7, diluted 1000x, and 40 μL of this dilution (containing ≈ 8 x10^4^ bacteria) were added to a microwell containing 100 μl 30% TSB, 25 μl 10mM SPB + 25 μl containing 100 μg of crypt lysate (fraction 4) or lysis buffer as a negative control. The total reaction volume was 200 μl. Cultures were incubated in a Wallace Victor plate reader (Perkin-Elmer Life Sciences, Boston, MA) at 37°C and O.D. 600 was measured and recorded every 15 minutes for 12 hours, and graphed. To evaluate the potential antimicrobial effects of recombinant mouse RELM-β, *C*. *rodentium* were serially diluted to 1000 bacteria and were then incubated with 10μl of either varying concentrations of RELM- or PBS as a control. Bacteria were allowed to grow overnight on LB-agar plates at 37°C after which the colony forming units were counted.

### CD4^+^ T cell reconstitution of *Rag1* ^*-/-*^ mice

Positively selected CD4+ T cells were purified from spleens and mesenteric lymph nodes of C57BL/6 mice using the Miltenyi MiniMACs purification apparatus (Miltenyi Biotec). In brief, spleens and MLNs were homogenized with a 1.0-mL syringe plunger and filtered and washed into a single-cells suspension. Cells were incubated with biotinylated anti-CD4 antibody (1:200, UBC) followed by incubation with streptavidin coated magnetic beads (1:30, Miltenyi) and run through magnetic columns. 2 x 10^5^ CD4+ cells were then injected into *Rag1* ^*-/-*^ mice via tail vein injection and left for 8 weeks, as previously described [13].

### FACS analysis

Colons were resected, longitudinally opened and washed in PBS containing 50ug/ml gentamycin. The colons were cut into 0.5-1cm pieces and incubated in HBSS solution containing 5%FBS, 2mM EDTA (Sigma), 1mM DTT (Sigma) and 10 mM Hepes (Sigma) for 30 min at 37°C. After vortexing briefly, the intestines were passed through a 100μm cell strainer to remove intraepithelial lymphocytes. The sections were then incubated in 5ml of RPMI medium containing 5% FBS, 1.5mg/ml of collagenase VIII (Sigma) and 0.1mg/ml DNAse (Sigma) for 1h at 37°C in a shaker. After vortexing for 1min, supernatants were filtered in a 70μm cell strainer and then resuspended in a medium. Further enrichment of lamina propria lymphocytes was done by the Percoll gradient method where the pelleted cells were resuspended in 8ml of 40% Percoll (Sigma) made in PBS which was layered on top of 4ml of 80% Percoll prepared in DMEM medium. Purified lymphocytes were collected at the interphase ring visible after centrifuging at 2000 rpm for 20min (with no braking). Subsequent FACS analysis was performed on freshly isolated lamina propria lymphocytes to identify the CD4+ve T cell populations. Briefly, 5x10^5^cells/well were fixed and permeabilized for 30min at room temperature using fixation/permeabilization concentrate (Ebioscience). Cells were then labelled with FITC and PE conjugated to anti-mouse CD4- (Ebioscience) and CD8 (Ablab) antibodies respectively. Cells were analyzed by using BD FACSDiva machine and data were analyzed by using FlowJo (Tree star) software. At least 30,000 events were counted for each sample.

### Transwell migration assays

4x10^5^ purified CD4^+^ T cells in 50μl of RPMI (Gibco) complete medium (10% FBS, 10nM non-essential amino acids, 10nM sodium pyruvate, 50μM β-mercaptoethanol and 50U/ml Penicillin-streptomycin) were placed in upper chamber of Transwells containing permeable polyester membrane (0.4 μm pores). In the lower chamber, 150μl of RPMI complete medium containing different concentrations of mouse recombinant RELM-β, 100 μg/ml BSA, 100ng/ml CCL4 or 100ng/ml denatured RELM-β was placed and incubated at 37°C for 4h. The number of cells transmigrated into the bottom chamber were counted using hematocytometer for each treatment condition.

### *In vitro* IEC proliferation

Mouse intestinal carcinogenic epithelial cells (CMT93) obtained from ATCC were cultured in high-glucose DMEM medium (Gibco) containing 10% FBS, 1x MEM and 5mM HEPES and 50U/ml penicillin-streptomycin. 5x10^4^ cells/well were grown on sterile glass coverslips in a 12-well plate (Corning) in a humid incubator at 37°C and 5% CO_2_. After reaching 50% confluency, the cells were treated for 24hr with 100ng/ml recombinant mouse IL-22 (R&D Biosystems) or RELM-β (Peprotech). Cells were subsequently fixed in ice-cold paraformaldehyde for 15 min followed by blocking with 1% BSA for 30min and incubation with rabbit derived anti-Ki67 (Thermo Scientific) for 2hr. After washing 3X with PBS, cells were incubated in dark for 1hr with secondary antibody conjugated to Alexafluor 568. After removal of secondary antibodies, cells were washed 3X with PBS followed by mounting in Vectashield (Vector Laboratories, Burlington, ON, Canada) on glass slides and screened with a Zeiss microscope.

### IL-22 neutralization studies and ELISA

IL-22 neutralization studies were carried out in *C*. *rodentium* infected C57BL/6 mice by injecting them with either neutralizing IL-22 antibodies (R&D systems), PBS or with an isotype matched control antibody (R&D systems) given on 0 and 6 DPI (100μg/mouse). Mice were euthanized at 8 DPI to collect colon tissues and then analyzed as above (including measuring IEC proliferation by Ki67 staining). For IL-22 ELISAs, colon and cecal tissues obtained from *C*. *rodentium* infected C57BL/6, *Retnlb* ^*-/-*^ and *Tcrβ* ^*-/-*^ mice either treated with rRELM-β or left untreated were extracted and the luminal contents were removed by washing with PBS. The pre-weighed tissues were cut into 0.5-1cm sections and incubated in DMEM containing 10% FBS supplemented with 50U/ml Penicillin-streptomycin and 50μg/ml gentamicin for 18 hours at 37°C in 5% CO_2_. Supernatants were collected by centrifugation at 12,000g for 10 min and the amount of IL-22 secreted into the supernatant was measured using the mouse IL-22 ELISA MAX (BioLegend, San Diego, CA) according to the manufacturer’s protocol.

### Statistical analysis

Statistics was carried out using GraphPad Prism Software Version 5 (GraphPad Software, San Diego, California, USA). Significance was calculated using the unpaired two-tailed Student’s t-test, Mann-Whitney test and One-way ANOVA followed by Bonferroni or Tukey-post hoc test as appropriate. *P* < 0.05 was considered significant and results expressed as the mean ± standard error (SEM).

## Supporting Information

S1 Fig RELM-β is strongly induced during natural infection. (A) Colonization of *C*. *rodentium* at 4 and 7 days post exposure (DPE) in uninfected C57BL/6 mice cohoused with mice shedding 10^8^ cfu/gram stool. Each data point represents 1 mouse in which the infection was transmitted. n = 4/timepoint. (B) qPCR of RELM-β gene (*retnlb*) expression in the distal colonic tissues of uninfected (n = 5) and infected (7 DPE; n = 4) mice. Results show mean of 4–5 mice/group. Error bars = SEM. *** P < 0.0001, Students *t*-test.
(TIF)

S2 Fig Further characterization of *C*. *rodentium* infection and large intestinal responses in the absence of RELM-β. (A) Resected large intestines of indicated mice, representative of n = 4 mice/group. Arrows, focal bloody ulcer. (B) Representative H&E staining of distal colons shown in “A”. Arrow, ulcer. (C) H&E stained cecal sections from uninfected vs. infected (10 DPI) WT and *Retnlb* ^*-/-*^ mice. Results are representative of n = 5 mice/group. Original magnification = 100X. Scale bar = 100 μm. (D) Quantitative PCR for cytokine gene expression in the cecal and rectal tissues of uninfected or 10 DPI BL/6 and *Retnlb* ^*-/-*^ mice. Results represent mean of 4–11/group. Error bars = SEM. *P ≤ 0.05 *Retnlb* ^-/-^ 10 DPI vs. uninfected BL/6 and *Retnlb* ^-/-^, 1-way-ANOVA with Dunn’s multiple comparison test. (E) Enumeration of *C*. *rodentium* in the cecal tissue and luminal compartments of the cecum of *Retnlb* ^*-/-*^ and C57BL/6 mice following infection. Results show means of 3–4 (2 and 4 DPI) and 6–11 (6–10 DPI) animals. **P = 0.0012 for 10 DPI; ***P = 0.0016 for 6 DPI, ^##^ *P* = 0.0016 for 10 DPI, *Retnlb* ^-/-^ vs. C57BL/6. (F) *C*. *rodentium* staining in mouse cecal tissues (10 DPI) showing penetration of crypts (arrows). Original magnification = 200X. (G) Immunostaining for the *C*. *rodentium* T3SS effector Tir at 8 DPI demonstrates large numbers of *C*. *rodentium* directly infecting epithelial cells near the base of crypts in *Retnlb* ^*-/-*^ mice whereas *C*. *rodentium* only infects superficial epithelial cells at the top of crypts in C57BL/6 mice. All results are representative of 2–3 independent experiments.
(TIF)

S3 Fig Severity of *C*. *rodentium*-induced disease in *Retnlb* ^*-/-*^ mice is partially dependent on the virulence factor EspF. (A) Measurement of bodyweight following infection wild-type (wt) or *ΔespF C*. *rodentium*. Each data point shows means of 4 mice/group. *P <0.05, vs. *Retnlb* ^*-/-*^ (wt *C*. *rodentium*) and C57BL/6 (*ΔespF C*. *rodentium*), Bonferroni post-test of 2-way ANOVA. Results represent 2 independent experiments, 4 mice/group. (B) Macroscopic analysis of large bowel of wt- and *ΔespF C*. *rodentium*-infected mice (10 DPI). (C) H&E staining of rectal tissues of mice described in (A) and (B). Original magnification = 100X. Scale bar = 100 μm. (D) wt and *ΔespF C*. *rodentium* enumeration within luminal compartments of infected colons (top) and ceca at 10 DPI. Each data point = 1 mouse and means are data pooled from 2 independent infections. Error bars = SEM. *P < 0.05; **P < 0.01; ***P < 0.001; ns = non-significant, Mann-Whitney *U* test.
(TIF)

S4 Fig No observable defects in the antimicrobial capacity and pro-inflammatory cytokine secretion in the colons of *Retnlb* ^*-/-*^ mice. (A) qPCR analysis of expression of genes known to regulate *C*. *rodentium* burdens (*inos*, *cnlp*) or host survival (*reg3g*) in the rectal tissues of uninfected vs infected (10 DPI) mice. Bars show the means of 3–4 mice/group. Error bars = SEM. *P ≤ 0.05 *reg3g* expression *Retnlb* ^-/-^ 10 DPI vs. uninfected C57BL/6, 1-way-ANOVA with Dunn’s multiple comparison test. (B) Growth curves of *C*. *rodentium* exposed to crypt lysates from C57BL/6 or *Retnlb* ^*-/-*^ mice. Crypt lysis buffer = control. (C) Percent survival of *C*. *rodentium* exposed to varying concentrations of RELM-β or PBS as a control. The experiment was performed twice. (D) qPCR analysis of cytokine gene expression within colonic tissues of uninfected or infected (10 DPI) mice. n = 4–11 mice/group, pooled from 2 separate infections. Error bars = SEM *P ≤ 0.05 *Retnlb* ^-/-^ 10 DPI vs. uninfected BL/6, 1-way-ANOVA with Dunn’s multiple comparison test. (E) Immunostaining for macrophages (F4/80 staining) and neutrophils (MPO) at 8 DPI. Original magnification = 200X. Scale bar = 50 μm. Representative of n = 4/ group.
(TIF)

S5 Fig Baseline IEC proliferation is not altered in the absence of RELM-β; RELM-β expression is not affected by presence or absence of CD4^+^ T cells. (A) Representative immunostaining for baseline proliferation in distal colons. (B) Quantitation of Ki67^+^ cells/crypt. Results show the mean of 20–30 well-oriented crypts counted over 4 random images/mouse. (C) RELM-β staining (red) of *C*. *rodentium* infected colons (10 DPI) of *Rag1* ^*-/-*^ mice reconstituted with PBS (controls) or with CD4^+^ T cells. Images are representative of 4 mice/group.
(TIF)

S6 Fig No overt differences in the responses of adaptive immune cells in infected C57BL/6 and *Retnlb* ^*-/-*^ mice. ELISA of (A) IFNγ and (B) IL17A secretion from splenocytes isolated from infected C57BL/6 and *Retnlb* ^*-/-*^ mice (10 DPI) after stimulation with media or *C*. *rodentium*-derived antigen. Results represent mean of at least 3 animals/group. Error bars = SEM.
(TIF)

S7 Fig T-cells are an important source of IL-22 during *C*. *rodentium* infection. (A) Ki67 positive cells in CMT-93 cells treated with rRELM-β (100ng/ml). (B) qPCR analysis for *Il-22* transcription in colon tissues obtained from *C*. *rodentium* infected *Tcrβ* ^*-/-*^ mice and C57BL/6 mice at 8 DPI (C) Supernatants obtained from the above mentioned colon tissues were assayed for IL-22 protein levels by ELISA. Results represent the means of 5 animals/group. Error bars = SEM, ***P < 0.0001 Students *t*-test.
(TIF)

S8 Fig Intraperitoneal delivery of recombinant murine RELM-β does not ameliorate disease in *Retnlb* ^*-/-*^ mice. (A) Resected large intestines of indicated mice. Arrows, focal ulcers. (B) Body weights of *Retnlb* ^-/-^ mice following rRELM-β or PBS injection. Error bars = SEM. (C) and (D) Enumeration of *C*. *rodentium* burdens. Each data point represents one animal. (Note: only two are for shown for colon lumen in control group due to lack of stool content in one of the mice). Results were determined from n = 3/group.
(TIF)

S1 Table Primer sets and PCR conditions used in this study. All PCR reactions had an initial denaturing step of 95°C for 3–5 minutes before commencement.
(DOCX)

S1 References Supporting information references. (DOCX)
