# Supplementary figures and images for: Goblet Cell Derived RELM-β Recruits CD4+ T Cells during Infectious Colitis to Promote Protective Intestinal Epithelial Cell Proliferation
Source: PLoS Pathog. 2015 Aug 18;11(8):e1005108. doi: 10.1371/journal.ppat.1005108 (PMC4540480; doi:10.1371/journal.ppat.1005108)

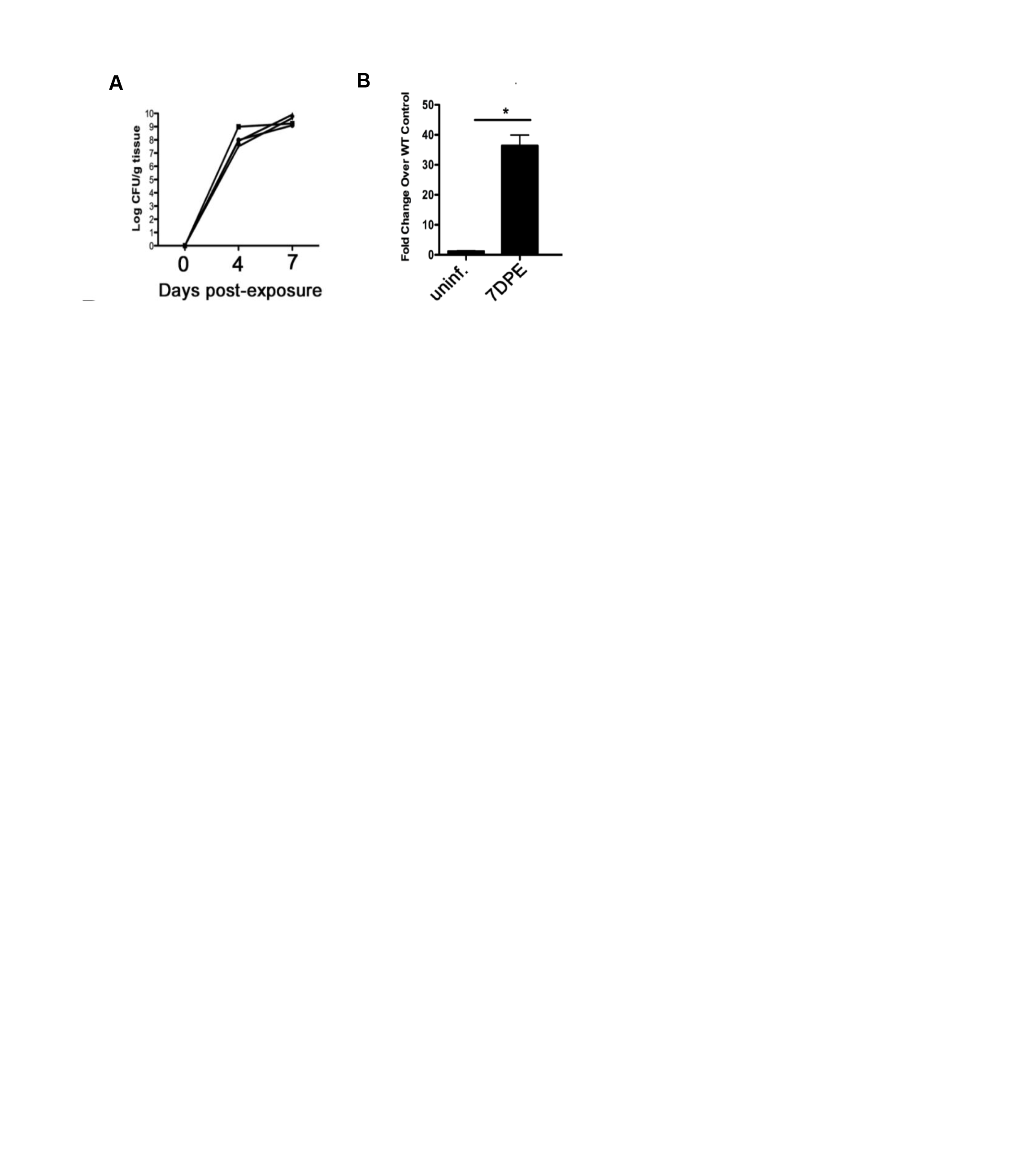

Supplement: S1 Fig — (A) Colonization of C. rodentium at 4 and 7 days post exposure (DPE) in uninfected C57BL/6 mice cohoused with mice shedding 108 cfu/gram stool. Each data point represents 1 mouse in which the infection was transmitted. n = 4/timepoint. (B) qPCR of RELM-β gene (retnlb) expression in the distal colonic tissues of uninfected (n = 5) and infected (7 DPE; n = 4) mice. Results show mean of 4–5 mice/group. Error bars = SEM. *** P < 0.0001, Students t-test. (TIF) [file ppat.1005108.s001.tif]

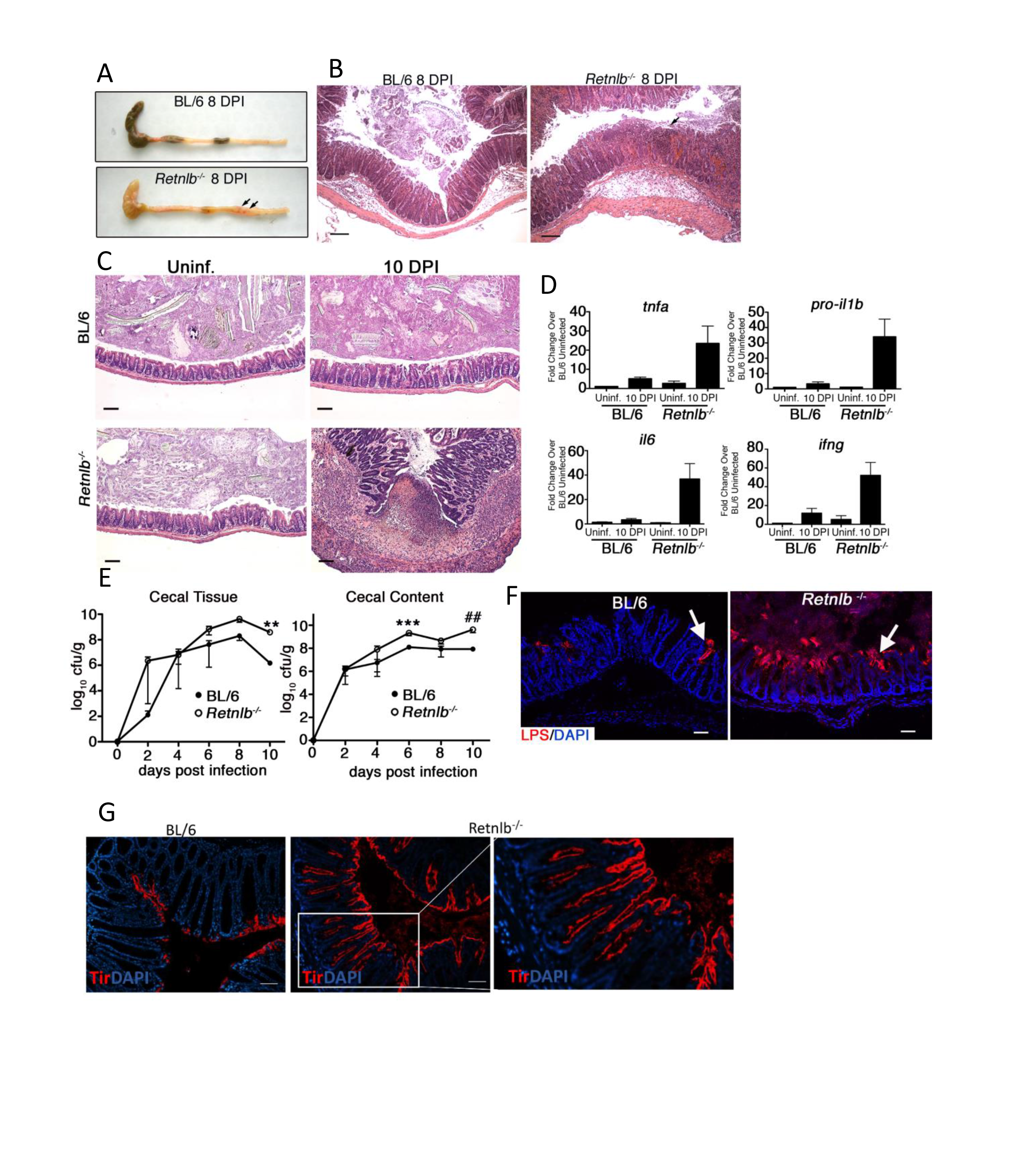

Supplement: S2 Fig — (A) Resected large intestines of indicated mice, representative of n = 4 mice/group. Arrows, focal bloody ulcer. (B) Representative H&E staining of distal colons shown in “A”. Arrow, ulcer. (C) H&E stained cecal sections from uninfected vs. infected (10 DPI) WT and Retnlb -/- mice. Results are representative of n = 5 mice/group. Original magnification = 100X. Scale bar = 100 μm. (D) Quantitative PCR for cytokine gene expression in the cecal and rectal tissues of uninfected or 10 DPI BL/6 and Retnlb -/- mice. Results represent mean of 4–11/group. Error bars = SEM. *P ≤ 0.05 Retnlb -/- 10 DPI vs. uninfected BL/6 and Retnlb -/-, 1-way-ANOVA with Dunn’s multiple comparison test. (E) Enumeration of C. rodentium in the cecal tissue and luminal compartments of the cecum of Retnlb -/- and C57BL/6 mice following infection. Results show means of 3–4 (2 and 4 DPI) and 6–11 (6–10 DPI) animals. **P = 0.0012 for 10 DPI; ***P = 0.0016 for 6 DPI, ## P = 0.0016 for 10 DPI, Retnlb -/- vs. C57BL/6. (F) C. rodentium staining in mouse cecal tissues (10 DPI) showing penetration of crypts (arrows). Original magnification = 200X. (G) Immunostaining for the C. rodentium T3SS effector Tir at 8 DPI demonstrates large numbers of C. rodentium directly infecting epithelial cells near the base of crypts in Retnlb -/- mice whereas C. rodentium only infects superficial epithelial cells at the top of crypts in C57BL/6 mice. All results are representative of 2–3 independent experiments. (TIF) [file ppat.1005108.s002.tif]

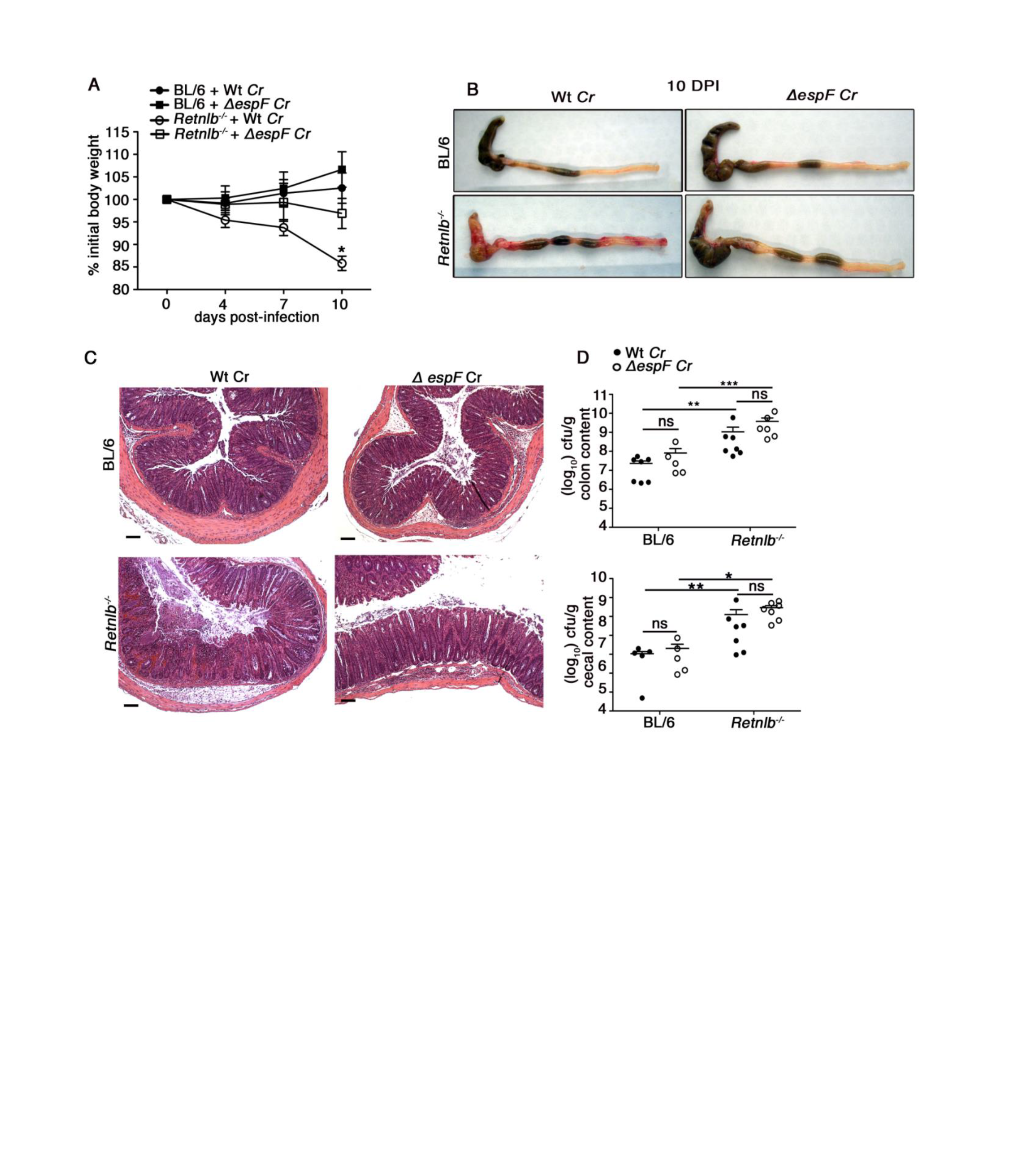

Supplement: S3 Fig — (A) Measurement of bodyweight following infection wild-type (wt) or ΔespF C. rodentium. Each data point shows means of 4 mice/group. *P <0.05, vs. Retnlb -/- (wt C. rodentium) and C57BL/6 (ΔespF C. rodentium), Bonferroni post-test of 2-way ANOVA. Results represent 2 independent experiments, 4 mice/group. (B) Macroscopic analysis of large bowel of wt- and ΔespF C. rodentium-infected mice (10 DPI). (C) H&E staining of rectal tissues of mice described in (A) and (B). Original magnification = 100X. Scale bar = 100 μm. (D) wt and ΔespF C. rodentium enumeration within luminal compartments of infected colons (top) and ceca at 10 DPI. Each data point = 1 mouse and means are data pooled from 2 independent infections. Error bars = SEM. *P < 0.05; **P < 0.01; ***P < 0.001; ns = non-significant, Mann-Whitney U test. (TIF) [file ppat.1005108.s003.tif]

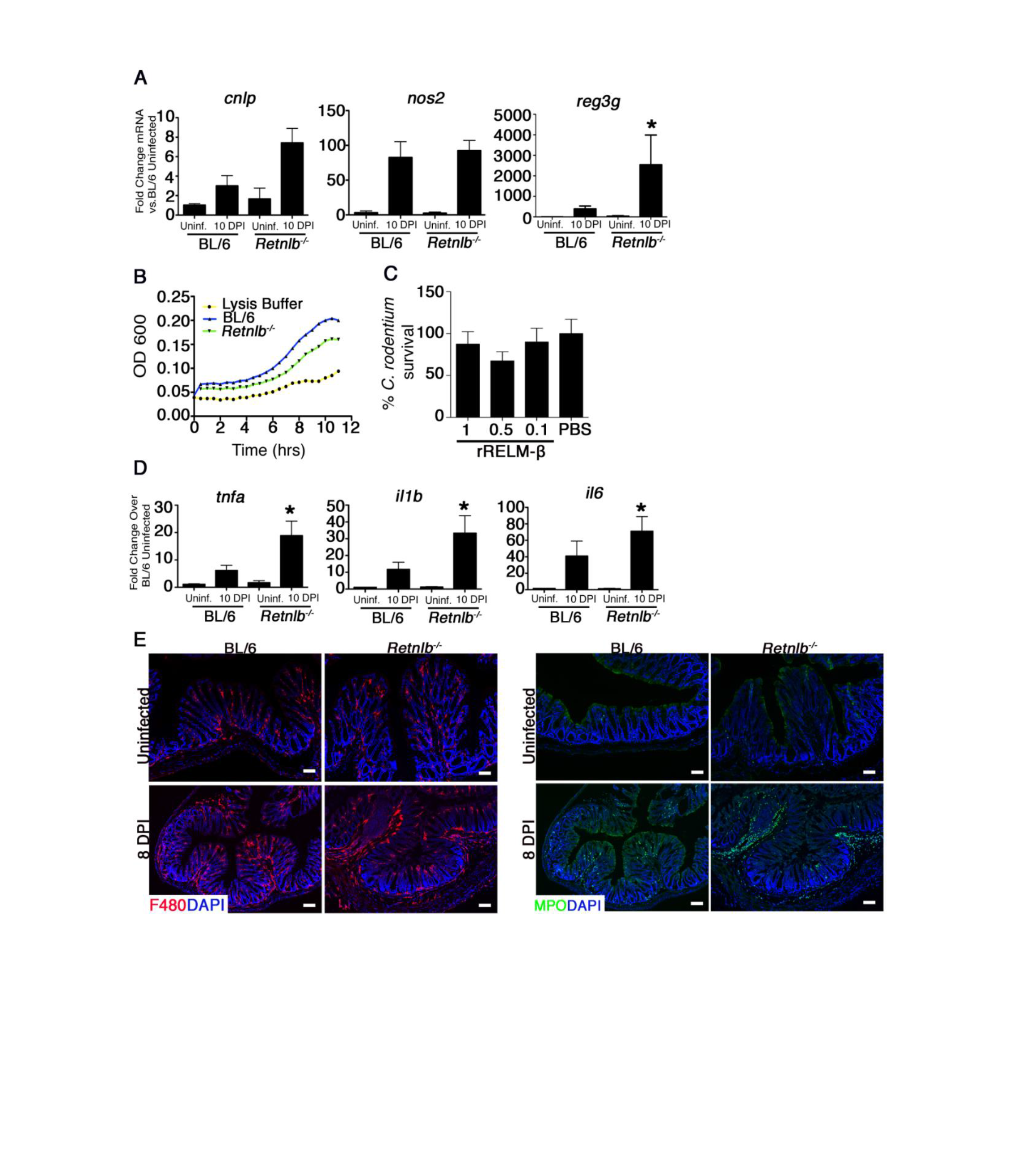

Supplement: S4 Fig — (A) qPCR analysis of expression of genes known to regulate C. rodentium burdens (inos, cnlp) or host survival (reg3g) in the rectal tissues of uninfected vs infected (10 DPI) mice. Bars show the means of 3–4 mice/group. Error bars = SEM. *P ≤ 0.05 reg3g expression Retnlb -/- 10 DPI vs. uninfected C57BL/6, 1-way-ANOVA with Dunn’s multiple comparison test. (B) Growth curves of C. rodentium exposed to crypt lysates from C57BL/6 or Retnlb -/- mice. Crypt lysis buffer = control. (C) Percent survival of C. rodentium exposed to varying concentrations of RELM-β or PBS as a control. The experiment was performed twice. (D) qPCR analysis of cytokine gene expression within colonic tissues of uninfected or infected (10 DPI) mice. n = 4–11 mice/group, pooled from 2 separate infections. Error bars = SEM *P ≤ 0.05 Retnlb -/- 10 DPI vs. uninfected BL/6, 1-way-ANOVA with Dunn’s multiple comparison test. (E) Immunostaining for macrophages (F4/80 staining) and neutrophils (MPO) at 8 DPI. Original magnification = 200X. Scale bar = 50 μm. Representative of n = 4/ group. (TIF) [file ppat.1005108.s004.tif]

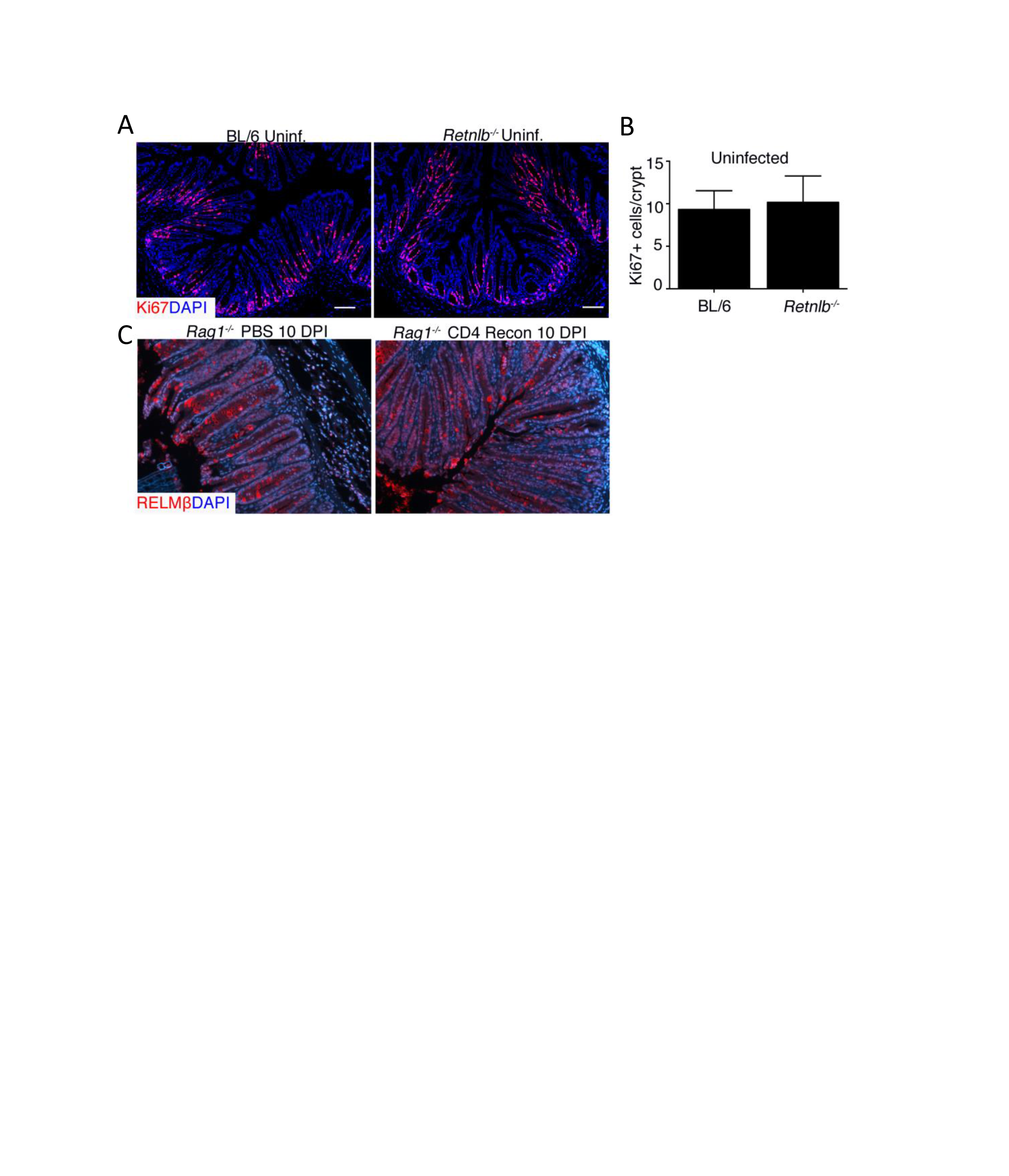

Supplement: S5 Fig — (A) Representative immunostaining for baseline proliferation in distal colons. (B) Quantitation of Ki67+ cells/crypt. Results show the mean of 20–30 well-oriented crypts counted over 4 random images/mouse. (C) RELM-β staining (red) of C. rodentium infected colons (10 DPI) of Rag1 -/- mice reconstituted with PBS (controls) or with CD4+ T cells. Images are representative of 4 mice/group. (TIF) [file ppat.1005108.s005.tif]

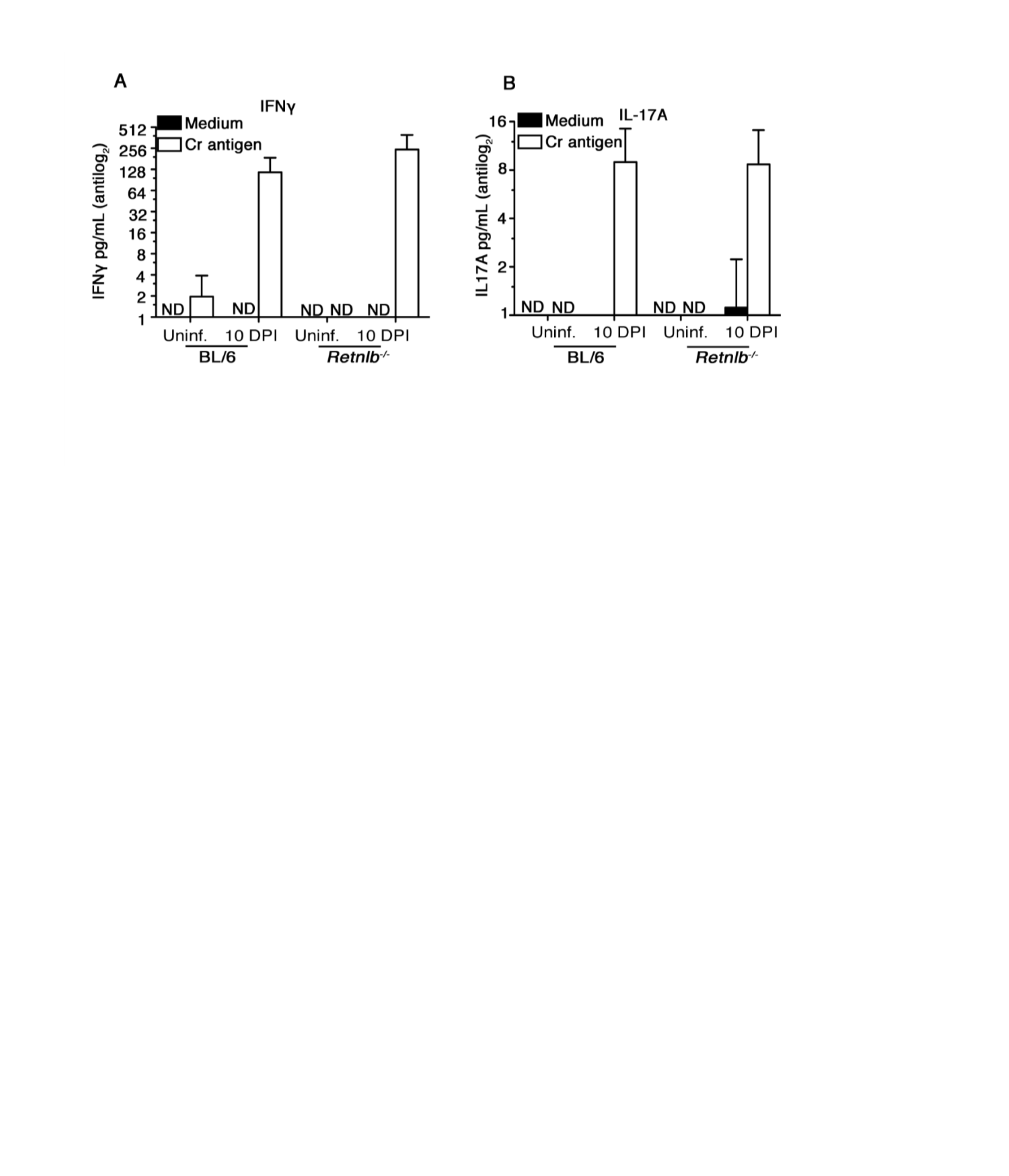

Supplement: S6 Fig — ELISA of (A) IFNγ and (B) IL17A secretion from splenocytes isolated from infected C57BL/6 and Retnlb -/- mice (10 DPI) after stimulation with media or C. rodentium-derived antigen. Results represent mean of at least 3 animals/group. Error bars = SEM. (TIF) [file ppat.1005108.s006.tif]

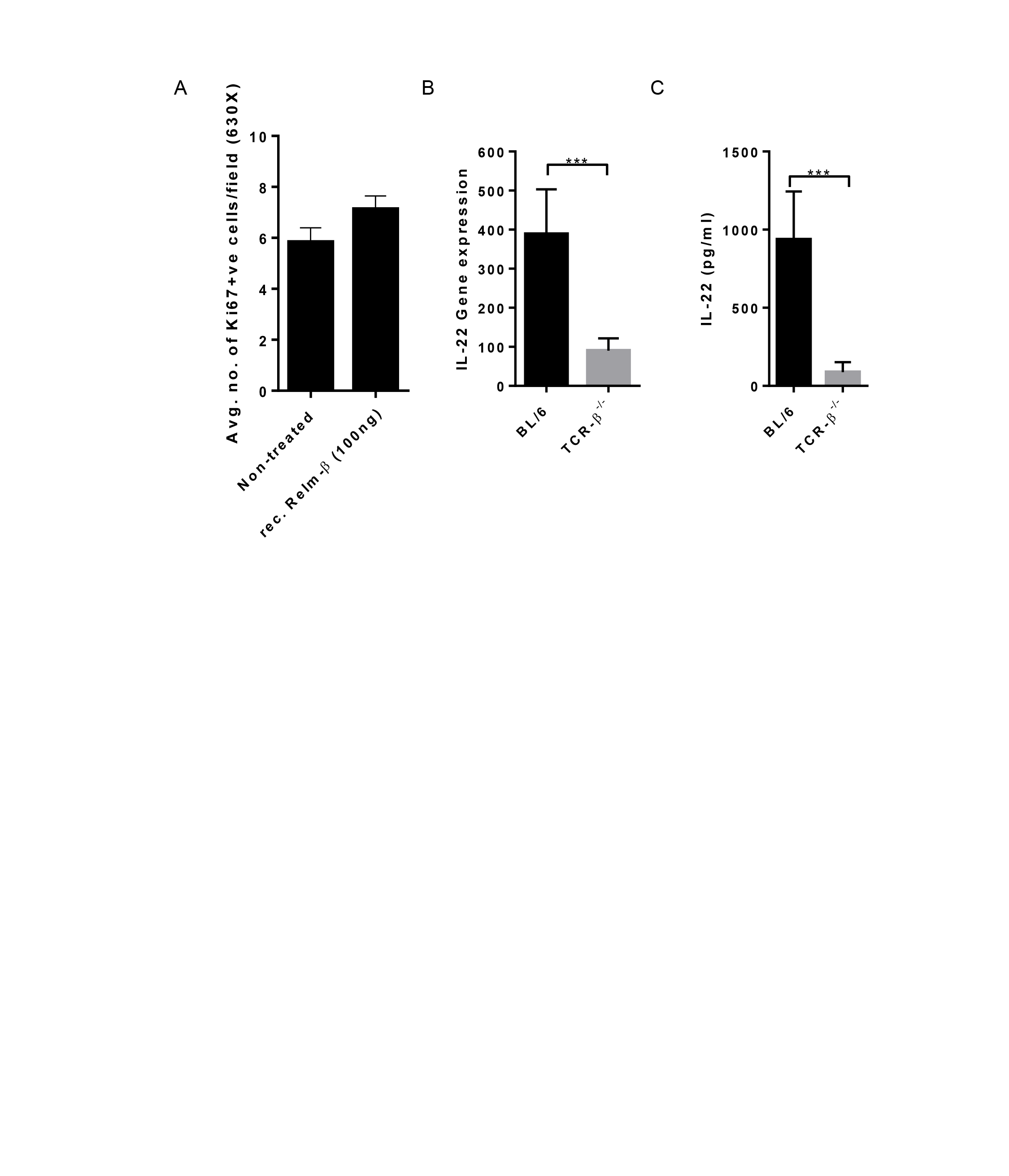

Supplement: S7 Fig — (A) Ki67 positive cells in CMT-93 cells treated with rRELM-β (100ng/ml). (B) qPCR analysis for Il-22 transcription in colon tissues obtained from C. rodentium infected Tcrβ -/- mice and C57BL/6 mice at 8 DPI (C) Supernatants obtained from the above mentioned colon tissues were assayed for IL-22 protein levels by ELISA. Results represent the means of 5 animals/group. Error bars = SEM, ***P < 0.0001 Students t-test. (TIF) [file ppat.1005108.s007.tif]

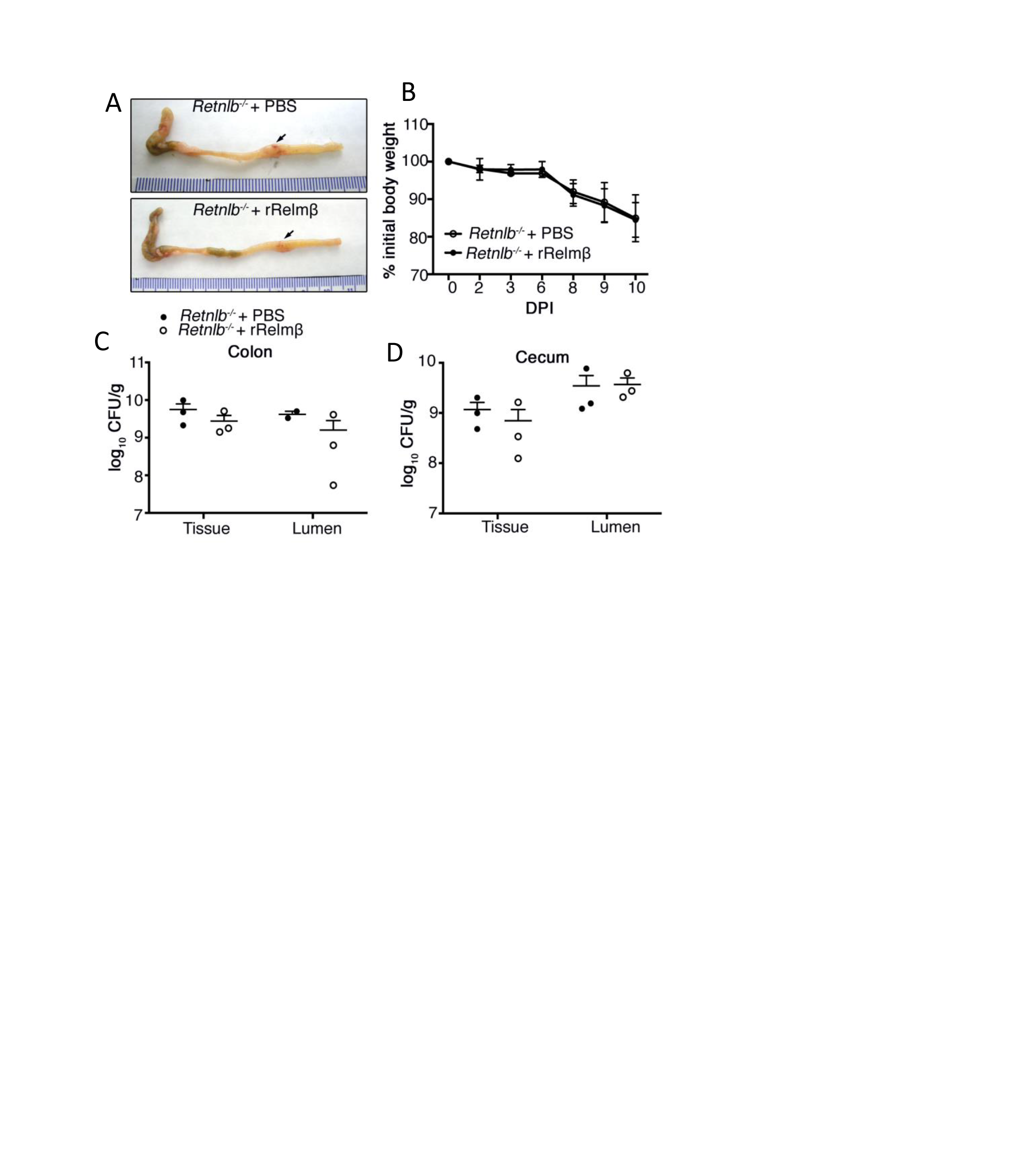

Supplement: S8 Fig — (A) Resected large intestines of indicated mice. Arrows, focal ulcers. (B) Body weights of Retnlb -/- mice following rRELM-β or PBS injection. Error bars = SEM. (C) and (D) Enumeration of C. rodentium burdens. Each data point represents one animal. (Note: only two are for shown for colon lumen in control group due to lack of stool content in one of the mice). Results were determined from n = 3/group. (TIF) [file ppat.1005108.s008.tif]
