# Supplementary material for: Goblet Cell Derived RELM-β Recruits CD4+ T Cells during Infectious Colitis to Promote Protective Intestinal Epithelial Cell Proliferation
Source: PLoS Pathog. 2015 Aug 18;11(8):e1005108. doi: 10.1371/journal.ppat.1005108 (PMC4540480; doi:10.1371/journal.ppat.1005108)
Supplement: S1 Table — All PCR reactions had an initial denaturing step of 95°C for 3–5 minutes before commencement. (DOCX) [file ppat.1005108.s009.docx]

| **Target mRNA** | **Primer Sets** | **PCR cycle conditions**  (denature/anneal/extend) |
| --- | --- | --- |
| RELM-β | Fwd: 5’-ccatttcctgagctttctgg-3’  Rev: 5’-agcacatccagtgacaacca-3’ | 94°C, 30s/ 55.5°C, 30s/ 72°C, 30s |
| Muc2 | Fwd: 5’-CTGACCAAGAGCGAACACAA-3’  Rev: 5’-CATGACTGGAAGCAACTGGA-3’ | 94°C, 30s/ 55°C, 30s/ 72°C, 45s |
| Tff3 | Fwd: 5’-CAGATTACGTTGGCCTGTCTCC-3’  Rev: 5’-ATGCTTGCTACCCTTGGACCAC-3’ | 94°C, 30s/ 60°C, 30s/ 72°C, 30s |
| TNF-α | Fwd: 5’- CATCTTCTCAAAATTCGAGTGACAA -3’  Rev: 5’- TGGGAGTAGACAAGGTACAACCC-3’ | 94°C, 30s/ 55°C, 30s/ 72°C, 45s |
| pro-IL-1β | Fwd: 5’-CAGGATGAGGACATGAGCACC-3’  Rev: 5’-CTCTGCAGACTCAAACTCCAC-3’ | 94°C, 30s/ 60°C, 30s/ 72°C, 45s |
| IL-6 | Fwd: 5’--3’GAGGATACCACTCCCAACAGACC  Rev: 5’--3’AAGTGCATCATCGTTGTTCAT | 95°C, 30s/ 60°C, 30s/ 72°C, 45s |
| IFN-γ | Fwd: 5’- TCAAGTGGCATAGATGTGGAAGAA -3’  Rev: 5’-TGGCTCTGCAGGATTTTCATG -3’ | 95°C, 30s/ 60°C, 30s/ 72°C, 30s |
| IL-22 | Fwd: 5’-ACCTTTCCTGACCAAACTCA-3’  Rev: 5’AGCTTCTTCTCGCTCAGACG-3’ | 94°C, 30s/ 58°C, 30s/ 72°C, 30s |
| iNOS | Fwd: 5’- TGGGAATGGAGACTGTCCCAG-3’  Rev: 5’- GGGATCTGAATGTGATGTTTG-3’ | 94°C, 30s/ 60°C, 30s/ 72°C, 30s |
| mCRAMP | Fwd: 5’- CTTCAACCAGCAGTCCCTAGACA-3’  Rev: 5’- TCCAGGTCCAGGAGACGGTA-3’ | 94°C, 30s/ 55°C, 30s/ 72°C, 30s |
| RegIII-γ | Fwd: 5’-tgcctatggctcctattgct-3’  Rev: 5’-cactcccatccacctctgtt-3’ | 94°C, 30s/ 58°C, 30s/ 72°C, 30s |
| mCCL8 | Fwd: 5’-AGCTGTGGTTTTCCAGACCAAGCA-3’  Rev: 5’-CACGCAGCCCAGGCACCAT-3’ | 94°C, 30s/ 60°C, 30s/ 72°C, 45s |
| mCXCL9 | Fwd: 5’-GCAGTGTGGAGTTCGAGGAACCC-3’  Rev: 5’-CCGAGTCCGGATCTAGGCAGGT-3’ | 94°C, 30s/ 60°C, 30s/ 72°C, 45s |
| mCCL25 | Fwd: 5’-CGTGCTGTGAGATTCTACTTCCGCC-3’  Rev: 5’-AGGGTGGCACTCCTCACGCT-3’ | 94°C, 30s/ 60°C, 30s/ 72°C, 45s |
| 18S rRNA | Fwd: 5’-GTAACCCGTTGAACCCCATT-3’  Rev: 5’-CCATCCAATCGGTAGTAGCG-3’ | 94°C, 30s/ 55°C, 30s/ 72°C, 45s |
| β-actin | Fwd: 5’-CAGCTTCTTTGCAGCTCCTT-3’  Rev: 5’-CTTCTCCATGTCGTCCCAGT-3’ | 94°C, 30s/ 55-60°C, 30s/ 72°C, 30s |

IFN-γ primers from ref. [23]; TNF-α primers, S1 ref.[1]; mCRAMP primers, S1 ref.[2]; iNOS primers, S1 ref.[3]; IL-17A primers, S1 ref.[4]; Tff3 primers, S1 ref [5]
