# Supplementary material for: Goblet Cell Derived RELM-β Recruits CD4+ T Cells during Infectious Colitis to Promote Protective Intestinal Epithelial Cell Proliferation
Source: PLoS Pathog. 2015 Aug 18;11(8):e1005108. doi: 10.1371/journal.ppat.1005108 (PMC4540480; doi:10.1371/journal.ppat.1005108)
Supplement: S1 References — (DOCX) [file ppat.1005108.s010.docx]

**S1 REFERENCES**

1. Hinz M, Schwegler H, Chwieralski CE, Laube G, Linke R, Pohle W, et al. Trefoil factor family (TFF) expression in the mouse brain and pituitary: changes in the developing cerebellum. Peptides 2004;5:827-832.

2. Nenci A, Becker C, Wullaert A, Gareus R, van Loo G, Danese S, et al. Epithelial NEMO links innate immunity to chronic intestinal inflammation. Nature 2007;446:557-561.

3. Rhee SJ, Walker WA & Cherayil BJ. Developmentally regulated intestinal expression of IFN-gamma and its target genes and the age-specific response to enteric Salmonella infection. J Immunol 2005;175:1127-1136.

4. Sugawara I, Yamada H, Li C, Mizuno S, Takeuchi O & Akira S. Mycobacterial infection in TLR2 and TLR6 knockout mice. Microbiol Immunol 2003;47:327-336.

5. Iimura M, Gallo RL, Hase K, Miyamoto Y, Eckmann L & Kagnoff MF. Cathelicidin mediates innate intestinal defense against colonization with epithelial adherent bacterial pathogens. J Immunol 2005;174:4901-4907.
